# Deciphering diversity at *er* loci for diversification of powdery mildew resistance in pea

**DOI:** 10.1038/s41598-022-19894-y

**Published:** 2022-09-26

**Authors:** Devinder K. Banyal, Himisha Dixit, Jaya Chaudhary, Anudeep B. Malannavar, Nisha Thakur

**Affiliations:** 1 Department of Plant Pathology, COA, CSKHPKV, Palampur, HP 176061 India; 2 Dr YSPUHF, KVK, Chamba, HP 17512 India

**Keywords:** Biotechnology, Plant biotechnology, Agricultural genetics, Plant sciences, Plant breeding

## Abstract

Agricultural biotechnology aims to scrutinize the field crops which feed half of the world’s population by improving their agronomic traits using various biotechnological tools. Pea- an important cash crop, rich in nutrients, but frequently infected with powdery mildew (fungal disease caused by *Erysiphe pisi*) that destroys the whole crop and causes economic loss for growers. We, therefore, targeted this research to find the pathogen-resistant pea lines and further decipher the diversity at *er* locus among resistant pea lines. Screening for resistant pea lines was done with *Erysiphe pisi* isolates (Genebank submission: KX455922.1) under the net house and greenhouse conditions. Molecular studies revealed that the *Erysiphe* resistant (*er1*) gene was present in 40 lines out of selected 50 pea lines and the mutational character was conferred up to 36 genotypes with 11 haplotype groups. The haplotype (gene) diversity (Hd) was found to be 0.5571 ± 0.099 SD and the nucleotide diversity (Pi) was 0.0160 ± 0.0042 SD Majority of resistant lines (67%) occurred in Hap-1, other remaining haplotypes (Hap 2–10) having 33% resistant lines, each showing characteristic nucleotide substitutions with respect to reference PsMLO1 gene; genotypes from these divergent haplotypes can be used in pea resistance breeding to avoid genetic homogeneity and genetic vulnerability.

## Introduction

During this global pandemic era, we are able to precise that the medical facilities become the priority for the life savior of the whole community. Although this is a universal talk, to feed the whole community, agriculture plays an equal and important role in the well-being and livelihood of the people globally. Peeping back into history to till this pandemic situation, we can elaborate on the role of various agricultural and horticultural crops to boost immunity against various diseases e.g. Turmeric, ajwain, ginger, garlic, among vegetables broccoli (anticancerous), lemon (vit c), and all green leafy vegetables (rich in iron). The crops are not only known for their nutritive value; but also provide economy to farmers. These crops are grown in the whole world according to their geographical and climatic conditions. If we start our journey from North-west Himalayas, we pay attention to the Pea crop (*Pisum sativum*) which has been grown for many centuries for green pods and grains to meet the nutritional demands and economic upliftment of the growers. Nutritionally, the pea crop comprises protein (25%), slowly digestive starch (50%), sugars (12%), amino acids, carbohydrates, vitamins (A and C), calcium and phosphorus^1^ along with lysine^2^. An interesting feature of this crop which increases its value as being a vegetable crop, it can be canned, frozen, dehydrated or dried and thus becomes a pulse crop. Being monumental, several preventive measures have been taken for crop protection which occurred due to biotic and abiotic stresses. Powdery mildew of pea is one of the common biotic stresses, which is caused by the *Erysiphe pisi* DC ex. Saint-Amans reduces the crop yield by up to 50 per cent by affecting the quality and quantity of green pods and dry seeds of pea^3,4^. Management of this drastic disease becomes a compulsion because the pathogen not only affected the grain and pods but also reduced pea foliage up to 33–69 per cent^5^. Banyal et al.^6^ developed disease-resistant cultivars by studying the pathogenic variability of *E. pisi* among various pea varieties. These resistant lines have *er* (*Erysiphe* resistant) locus having MLO gene (responsible for resistant mechanism in pea) which was detected using various molecular approaches. The present investigation, therefore, was carried out to find the presence of MLO gene among selected resistant cultivars of pea and decipher the diversity of *er* gene present among these resistant cultivars.

## Results

### Identification of test isolate

**Figure 1:**
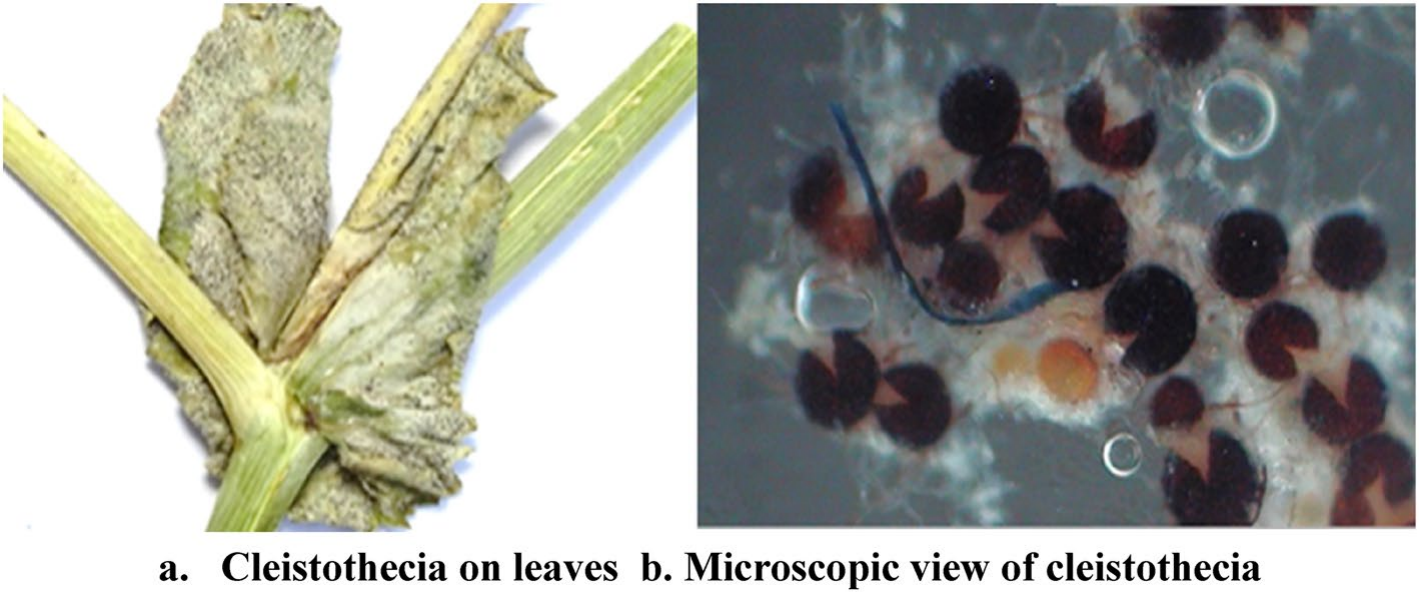
Cleistothecia of *Erysiphe pisi* causing pea powdery mildew are formed during sexual reproduction, appeared as spherical, gregarious, dark brown color, measuring about 87.5–133 µm in diameter and dispersed in the mycelial web.

**Figure 2:**
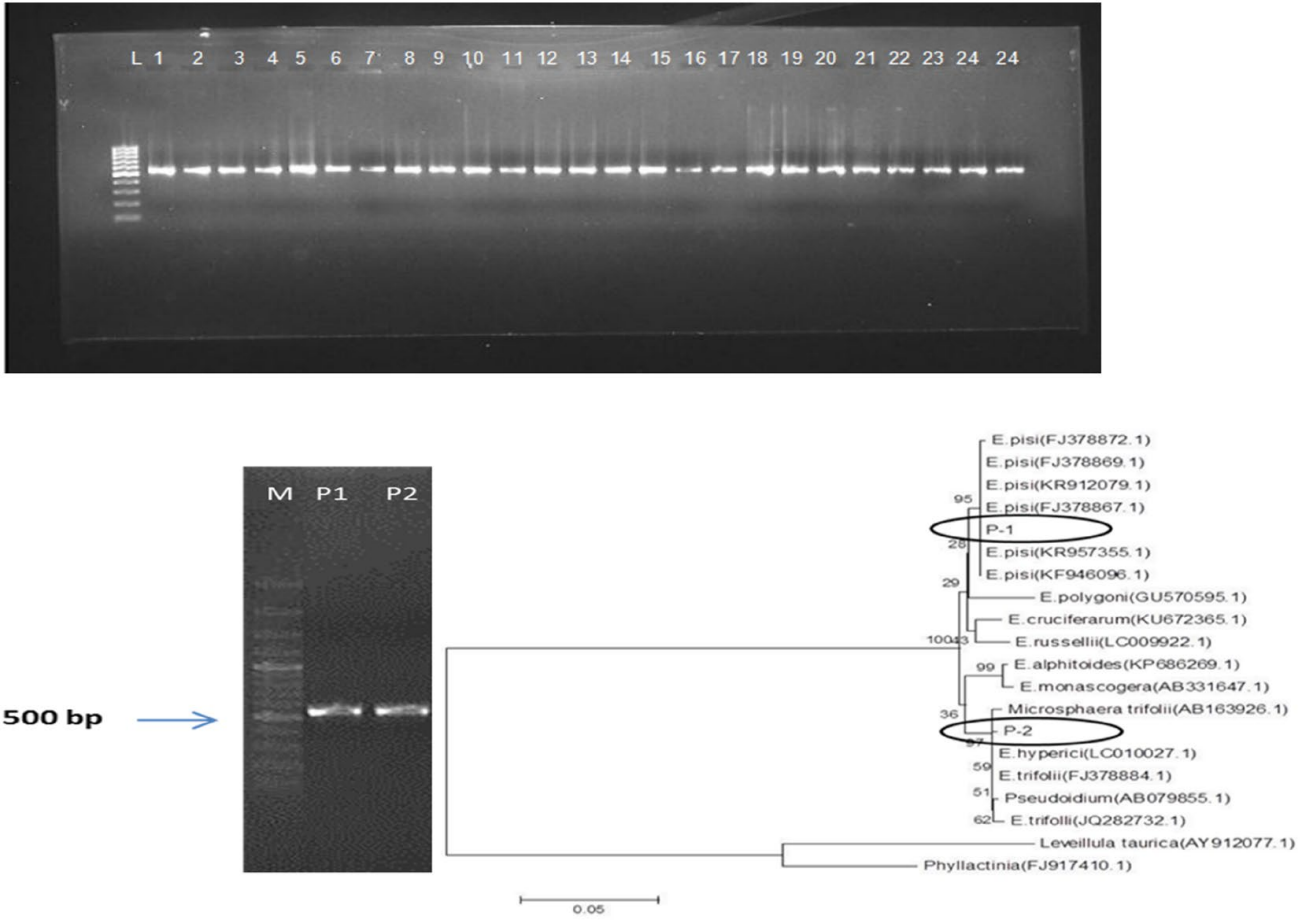
rDNA region amplified using *Erysiphe* specific primers-EryF(5’-TACAGAGTGCGAGGCTCAGTCG-3’) EryR (5’-GGTCAACCTGTGATCCATGTGACTGG-3’) (M: 1 Kb ladder; fungal isolates (P1-P24 UPPER) P-1 and P-2 along with tree phylogeny of P-1: *Erysiphe pisi*, P-2: *Erysiphe trifolii *(LOWER).

Morphological characteristics viz., hyphae, conidia, conidiophores, conidia size and conidiophore foot cells were studied on the detached leaves of the host using a stereo zoom microscope (Fig. 1; Table 1). Attanayake et al.^7^ described two groups of powdery mildew-infected pea pathogens in a combination of morphological and molecular characteristics. PCR amplification revealed an amplicon of approximately ~ 560 bp, (Fig. 2) which was further gel purified and lyophilized before sequencing. BLAST analysis of the sequences of test isolates P-1 (from pea) and P-2 (from clover) indicated that both the strains were placed in the phylogenetic lineage occupied by the genus *Erysiphe* along with species, *pisi* and *trifolii*, respectively (Fig. 2) (https://v3.boldsystems.org/index.php/IDS_BlastRequest). The 18S rRNA sequence strain has been deposited in the NCBI GeneBank (Accession numbers KX455922 and KX455923, respectively).

**Table 1:** Microscopic observations of powdery mildew causing fungal pathogen.

Characters	Test pathogen
Hyphae	Straight, branched, septate and hyaline
Conidia	Ellipsoid, cylindrical, ovoid shaped, hyaline and produced in chains
Conidiophores	Erect, straight, hyaline, arise vertically from the hyphae on the host surface
Foot cells	Cylindrical and decreased in width from base to the top
Cleistothecia	Spherical, gregarious, dark brown in colour and dispersed in mycelial web
Conidial (LXW)	25.5–52 × 11–18 µm
Conidiophores(LXW)	22–50 × 7–10.5 µm
Foot cells (LXW)	17.5–28 × 7-10 µm
Cleistothecia diameter	87.5–133 µm

### Screening for resistant pea lines

Previously, screening was done, and selected 3 resistant lines were crossed with JI-2302 (*er1*) and JI-2480 (*er2*) in 8 cross combinations viz., JI-2480 × Acacia, JI-2480 × PMR-10, JI-2480 × EC-381866–1, JI-2480 × Lincoln, JI-2302 × Acacia, JI-2302 × PMR-10, JI-2302 × EC-381866–1 and JI-2302 × Lincoln under net-house and greenhouse and description of infection were observed^6^. Resistant was governed in maximum cultivars due to the presence of the *er1* gene (Table 2). We, therefore, select the *er1* gene for further studies.

**Table 2:** Evaluation of F_1_ population to study the allelic relationship with known *er* genes against pea powdery mildew caused by *Erysiphe pisi.*

S. no	Cross	Infection type	Reaction type
1	JI-2480 (*er2*) × Acacia	2	Resistant (R)
2	JI-2480 × PMR-10	3	Susceptible (S)
3	JI-2480 × EC-381866–1	3	S
4	JI-2480 × Lincoln	4	S
5	JI-2302 (*er1*) × Acacia	3	S
6	JI-2302 × PMR-10	2	R
7	JI-2302 × EC-381866-1	2	R
8	JI-2302 × Lincoln	4	S

### Amplification studies

**Figure 3:**
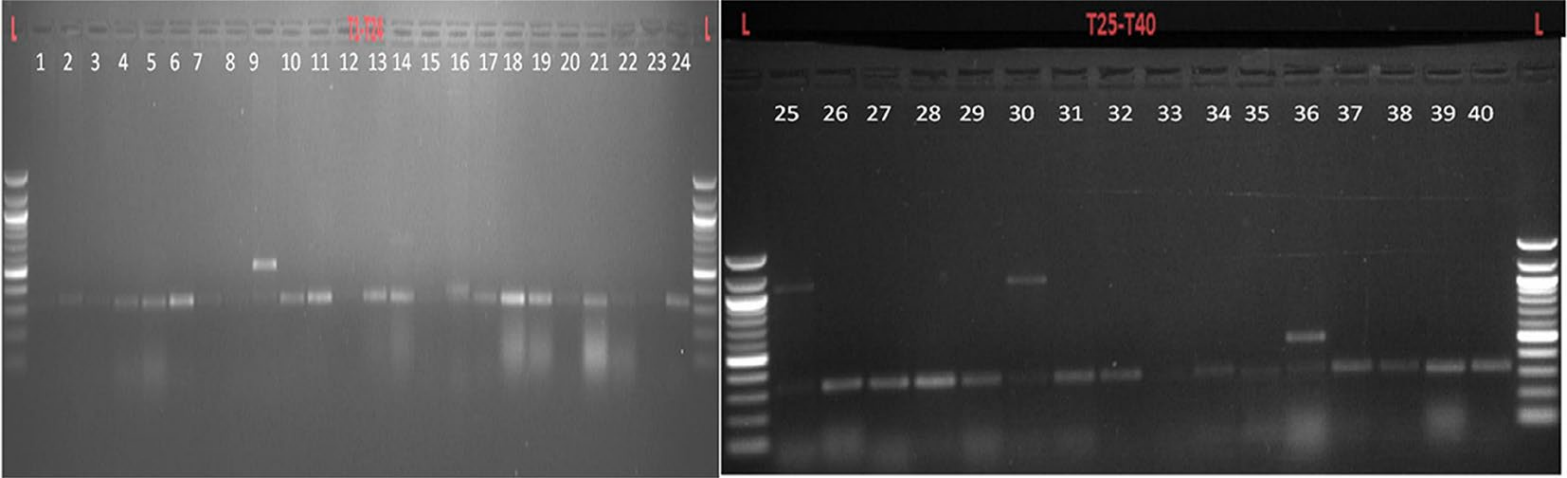
Amplification of DNA fragments (1–40) of selected pea lines. Amplification was possible with primer Primer PsMLO3F and PsMLO3R produced 40 amplicons of variable size (300–325 bp) in different genotypes used. L = Ladder (100 bp).

A total of 50 pea lines were used for RNA extraction (Fig. 3). cDNA prepared from RNA was further amplified by specific primers mentioned in the material and methods. To achieve this many times repeated PCRs were carried out in all the samples to standardize the protocol. Out of 50 lines, amplification was possible with primer 3F and 3R which produced 40 amplicons of variable size (300–325 bp) targeting that the *er1* gene was present only in these lines. (Table 3; Fig. 3).

**Table 3:** No. of amplified pea lines with primer PsMLO3F and R.

S. no	Pea genotypes
1	1P-1287
2	4P-995
3	5P-1395-2
4	7P-1301
5	8P-1805
6	9HFP-4
7	10P-1806
8	11P-1804
9	12P-1820
10	14P-144-10
11	15P-1280-4
12	16P-668-1
13	17P-1707
14	18P-48
15	19P-1610-9
16	20P-1436-9
17	21P-1813
18	22P-1377
19	23P-1422-1
20	24P1436-8
21	25P1610-2
22	26P-1506
23	27P-1811
24	28P-1440-20
25	29P-1516
26	30P-179
27	31IPF-99-25
28	32PKPMR-400
29	39DPP-139-3
30	40DPPMR-09-1
31	42LFP-517
32	43LFP-575
33	45LFP-571
34	48LFP-577
35	50PB-29B
36	51DPP-362
37	52ACACIA
38	55MR BIG
39	56KMNR-894
40	59DMR-11

### In silico analysis of gene sequences

**Figure 4:**
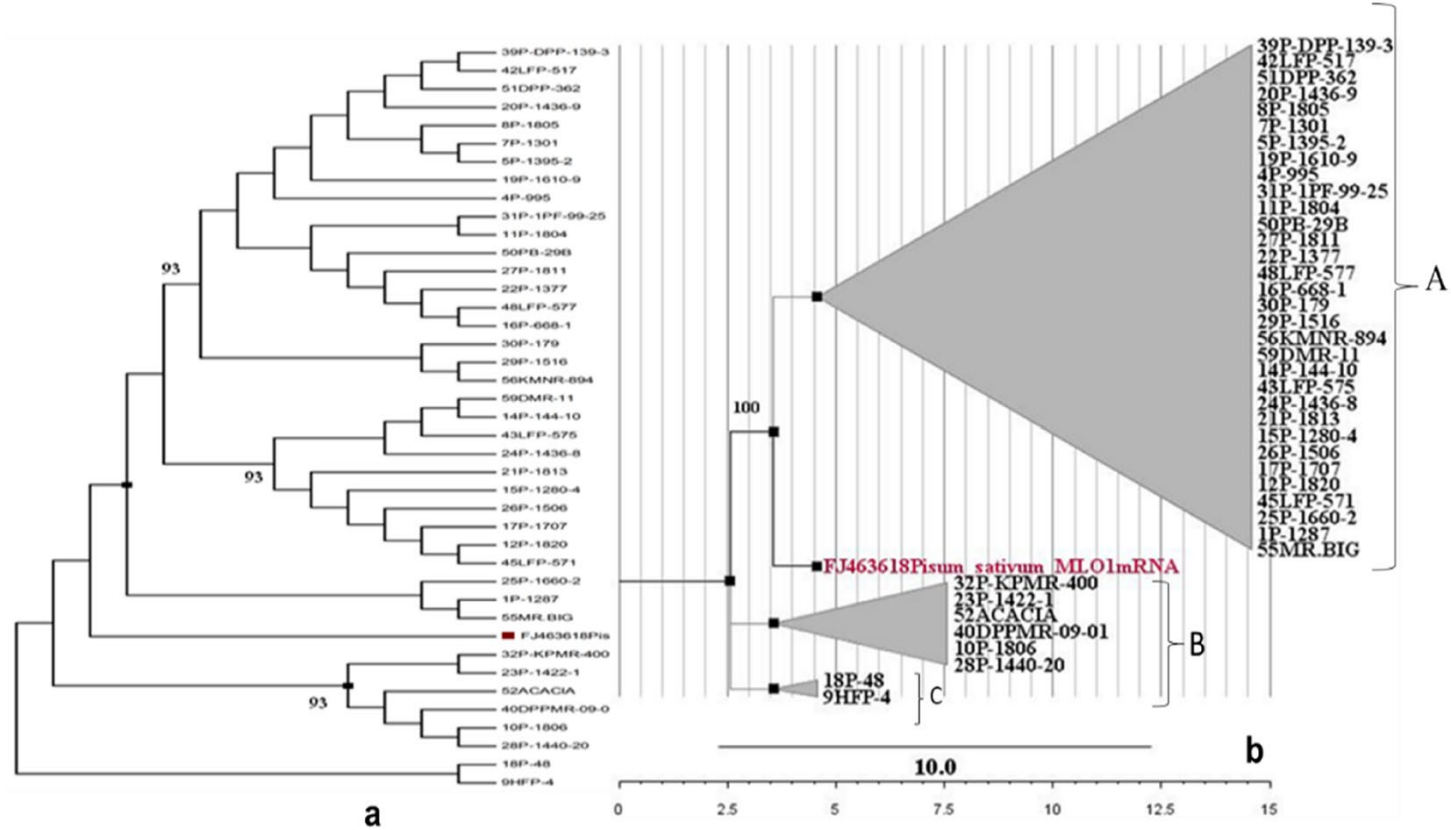
NJ tree based on MLO sequences. Posterior probabilities of the main clades above 0.93 (**a**) and bootstrap values in % (**b**) are indicated at the nodes (100). The designation includes pea lines number and name.

BLAST N search for the homology of all the sequences of *er1* gene corresponds to the gene present in Pisum sativum MLO1 (MLO1) mRNA, complete cds; homology queries values ≥ 90% and E values near 0 for Nucleotide Blast analysis. Phylogenetic analyses separated the pea accessions into 3 groups. The major group A constituted 32 accessions and the remaining 8 accessions were grouped as 6 and 2 in the B and C groups, respectively. The obtained tree was then saved to Newick format and the Fig Tree program was used for tree illustration (Fig. 4) (http://tree.bio.ed.ac.uk/software/figtree/).

### Number of haplotypes

A total of 11 haplotypes were obtained and the frequency of haplotypes ranged from 1 to 24. Hap-1 was the most abundant haplotype representing 24 genotypes including the reference genotype (FJ463618.1). The remaining haplotypes were represented by a single genotype except Hap- 4 which is represented by 3 genotypes (Table 4). The Hap-1 having 23 genotypes showed 100 per cent similarity with reference genotype (FJ463618.1), hence does not have any base substitution w.r.t PsMLO1, whereas, Hap-2, 3, 4, 5, 6, 7, 8, 9, 10 and 11 have 9, 5, 1, 6, 6, 16, 15, 14, 13 and 6 base substitutions, respectively.

**Table 4:** Frequency distribution of different PsMLO1 haplotypes detected amongst the resistant pea lines.

Hap#	Frequency	Lines	No of base substitution(s)w.r.t. reference PsMLO1gene
Hap-1	24 (23 + 1)	FJ463618.1, P-1287, P-1820, P-1516, P-144-10, P-1707, P-1610-9, P-1377, P-1506, DPMR-09-01, P-179, P-KPMR-400, P-1440-20, LFP-575, LFP-571, ACACIA, MR_BIG, P-1280-4, P-1422-1, P-1610-2, P-1436-8, P-1806, P-1813, DMR-11	0
Hap-2	1	P-1804	9
Hap-3	1	P-995	5
Hap-4	3	HFP-4 P-48 KMNR-894	1
Hap-5	1	P-668-1	6
Hap-6	1	P-1811	6
Hap-7	1	IPF-99-25	16
Hap-8	1	DPP-139-3	15
Hap-9	1	LFP-517	14
Hap-10	1	LFP-577	13
Hap-11	1	PB-29-B	6

### Polymorphic sites

**Figure 5:**
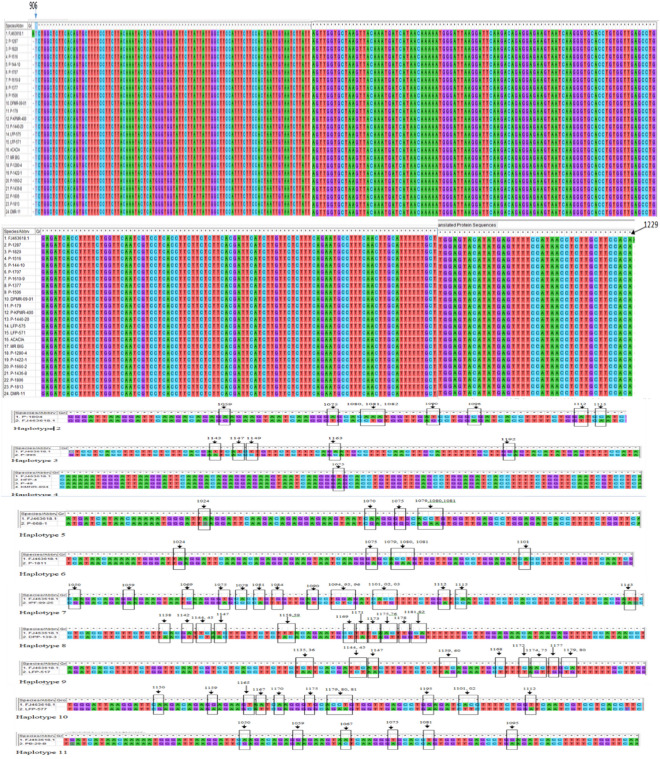
Sequence alignment of MLO gene (906–1229 bp) of powdery mildew resistant pea accessions belonging to Haplotype 1–11 with reference sequence FJ463618.1.

The analysis of polymorphic sites was carried out using DNAsp VI and a total of 36 sequences were used with a total of 198 variable sites. The 47 polymorphic sites included 18 Singleton variable sites, out of which 17 were with two variants and one was with three variants. There were 29 Parsimony informative sites out of which 26 were with two variants and three with three variants. The analysis of polymorphic sites was further studied in detail using multiple sequence alignment. Multiple sequence alignment was carried out in MEGA-(software) using the tool Clustal W^8^. Each haplotype of evaluated resistant pea genotypes was compared with the reference genotype for any site with replacement, deletion and addition (Fig. 5).

### Haplotype diversity and Tajima’s test

**Figure 6:**
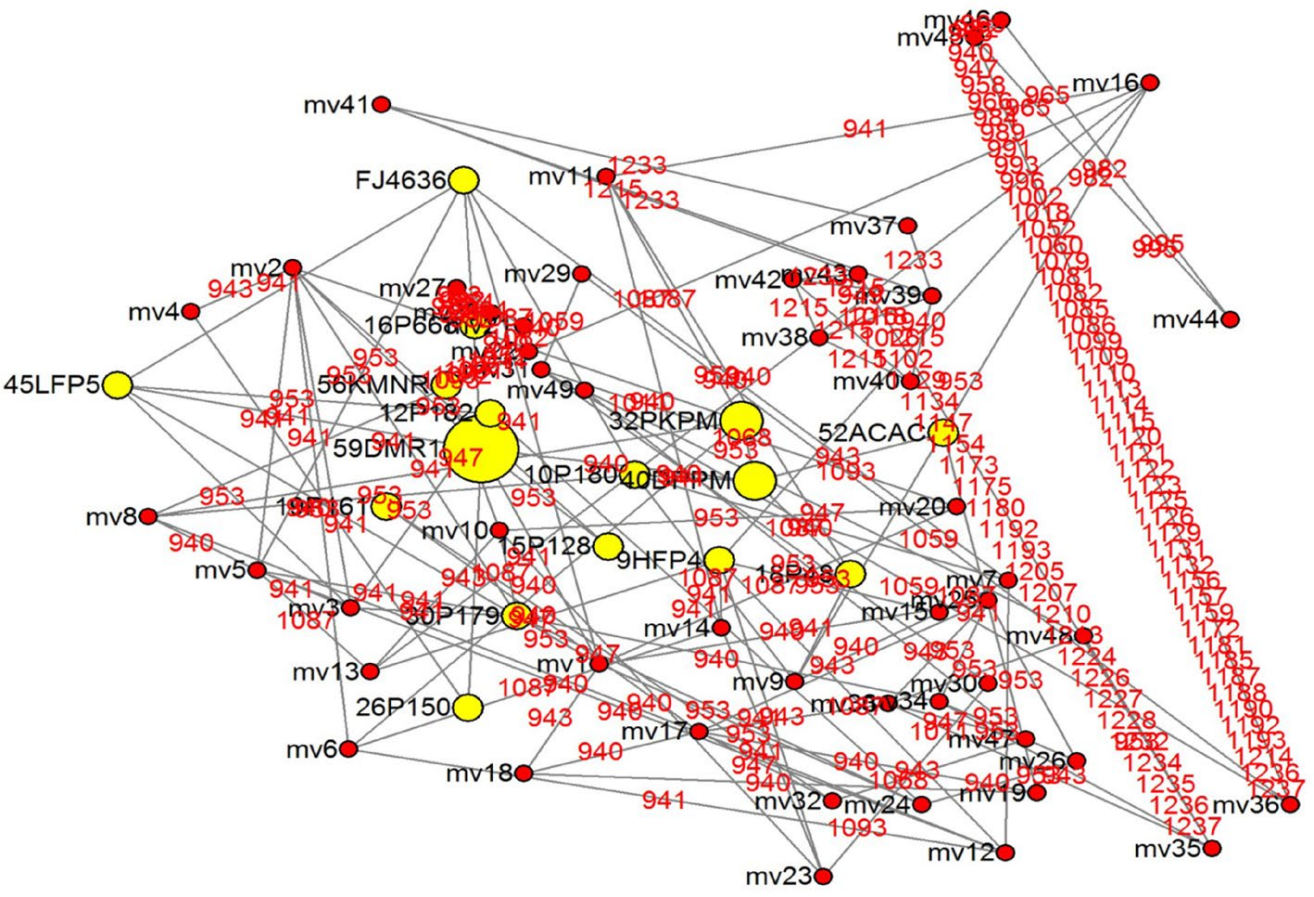
Median joining network with median vector (red color dots), mutated characters (red color taxa) and sequence frequency (yellow color dots).

Haplotype diversity was calculated using DNAsp VI where haplotype (gene) diversity was 0.5571 and Nucleotide diversity (per site) of 0.01606 (Table 5). Tajima’s test was also found to be statistically significant with Tajima's D-2.09021 at *P* < 0.05. A median-joining network inferred from 40 sets of sequences with 33 no. of active haplotypes was drawn. A value of zero was set for epsilon (e = 0) to calculate sparse networks quickly, or incrementally. The maximum no. of mutations (29) was found at character 941 and the least no. of mutations (1) were ranged at characters 992–1190 (Fig. 6).

**Table 5:** Haplotype diversity and Tajma test.

Haplotype (gene) diversity, Hd	0.5571
Standard Deviation of Haplotype diversity	0.099
Nucleotide diversity (per site), Pi	0.01606
Standard deviation of Pi	0.00462
Average number of nucleotide differences, k	5.20317
Number of polymorphic (segregating) sites	47
Total number of mutations (eta)	51
Tajima's d	− 2.09021

## Discussion

Pulses are majorly produced crops after cereals and field pea (*Pisum sativum)* is one of the widely cultivated crops^9^. To date, various research studies have been carried out for the management of powdery mildew in pea plants^10–12^. For long-term management and increase in yield production, it is required to develop genetically resistant crop plants^13^. Furthermore, the knowledge of germplasm resources and yield contributing characters are necessary to understand genetic diversity^14^. Knowing the importance of this vegetable crop by exploring the literature we, therefore, continued our investigation by determining the diversity in pathogen-resistant pea lines at er loci^15^, which were screened in vitro^16^. Collection and DNA profiling of powdery mildew causing pea pathogen^17,18^ corresponds to genus *Erysiphe* with species, *pisi* and *trifol*i, respectively^7,19,20^. The North-Western Himalayan region is the most prevalent hot spot of powdery mildew^16^. The sexual stage of a pathogen (cleistothecia) is frequently formed only in the dry temperate zone^21^ indicating the presence of the pathogenic virulence of *E. pisi* in Zone IV of the Himalayas. The study of pathogenic variability of *E. pisi* is the most important for breeding resistant varieties. The resistant varieties evolved against pea powdery mildew and become susceptible after a short time, indicating the existence and selection for the emergence of new *E. pisi* virulence. The study of pathogenic variability has therefore been required for the successful management of the disease through the identification, development, and deployment of resistance sources/varieties in a given geographic situation^6,22^. This could be helpful for us to a greater extent regarding breeding as well as in conservation aspects of the pea crop improvement program. Experimental results revealed that the screened cultivars (having *Erysiphe* *pisi* resistant gene) when crossed with carriers (JI-2302, JI-2480 (*er1* and *er2*) found to be governed by resistance by a single *er1* gene from the resistant carrier line JI-2302 (*er1*)^12,23–26^. Genes *er1* and *er 2* can be considered as major natural resistant bases^10,27–29^ against powdery mildew pathogen, thus introgressed into subsequent pea lines. Although *er2* gene which also conferred resistance, the maximum pea cultivars revealed the presence of *er1* gene conferring resistance against powdery mildew^12,25,26^. The *er1* gene obtained in resistant pea lines was further ensured using a molecular approach^30–33^. Past discoveries brought us the knowledge of naturally occurring random mutagenesis at Mildew Locus O (MLO) in many monocots/dicots^34^ which led to natural loss-of-function mutations. Reports suggest this mutation becomes beneficial for the host to terminate the fungal invasion at the first step, thus creating resistance. In the case of *Pisum sativum*, the PsMLO1 gene present in the crop provides a broad and durable resistance against the powdery mildew pathogen^35^, thus acting like a candidate gene to reveal the allelic diversity among resistant pea lines. Amplification with primers (PsMLO3FP and PsMLO3R) revealed that the candidates can be identified during the early stages of *E. pisi* infection^36^. In the cases of tomato^37^, barley^38^, pepper^39^ and grapevine^40,41^, resistant gene expression increased in response to the pathogen within the first 24 h and a peak of resistance was obtained around 6 h. Similarly, after infection of *E. pisi*, the resistance developed in pea lines after 4–8 days, which was observed morphologically, and resistant and susceptible rates were recorded^42^. During phylogenetic analysis, we found most of the *er1* gene sequences corresponded to the reference gene (*Pisum sativum* MLO1) of which the largest clade comprises of major group A corresponds to ≥ 90% of similarity with *Pisum sativum* MLO1 sequences^43,44^. The results were found to be in harmony with the results obtained by many collaborators^35,37^. Accession of pea lines in a major clade of group A (Fig. 4) having the MLO1 sequences (analog of *er-1* gene) can thus be used directly in future breeding programs. According to NIH (National Human Genome Research), haplotypes are allelic combinations (single /multiple) where the polymorphism found very close in between the genes thus inherited together without any recombination, subsequently used in genetic studies. Haplotype based approach used for identification of genetic divesity in bread wheat (*Triticum aestivum*) cultivars had been extensively used by many scientists^45^ thus become a useful technique in crop improvement programmes. We found a total of 11 haplotype groups where the frequency of haplotypes ranged from 1 to 24. Among these groups, Hap-1 was the most abundant haplotype, representing 23 genotypes, including the reference genotype (FJ463618.1), which revealed no base substitution w.r.t PsMLO1. The genotypes having the *er1* gene grouped in Hap-1 represent the resistant alleles passed from resistant carriers linked together without any substitutions. In the case of Hap-2, 3, 4, 5, 6, 7, 8, 9, 10 and 11, we found base substitutions of 9, 5, 1, 6, 6, 16, 15, 14, 13 and 6, respectively at the MLO locus, showing the diversity in the *er1* gene substitutions (Fig. 5). Each haplotype of evaluated resistant pea genotypes was compared with the reference genotype for any site with replacement, deletion and addition. The genotypes from these divergent haplotypes can be used in pea resistance breeding to avoid genetic homogeneity and genetic vulnerability. In case of the fruit crop divergent cultivars can be used for domestication of early and late maturing cultivars in lychee^46^. Statistical calculations revealed the haplotype diversity of 0.5571 ± 0.099 SD and nucleotide diversity (Pi) of 0.0160 ± 0.0042 SD. A low value of nd (0.01606) and a negative value of Tajima's D -2.09021 at *P* < 0.05 (statistically significant) revealed that these resistant lines can’t be affected by environmental conditions. Nucleotide diversty (nd) of the cultivated varieties of Korean rice accessions (weedy = 0.0102, landrace = 0.0093, and bred = 0.0066) was found to be lower, revealed no reduction in diversity during domestication^47^. To illustrate the molecular data for intraspecific studies, various haplotype networks were previously used ^48^. In simple terms, these networks provide insight into the population structure, migration and new species creation^49^. Here, we draw a median-joining network (MJN) of haplotypes with mutated characters (Fig. 6). Literature supported that the MJ method required the least no. of mutations, which yielded a good genealogy^50^. Also, this MJ approach functioned properly when haplotypes were comparatively distant^51^ and displayed a good network construction under low substitution rates^52^. Kong et al.^53^ discussed the use of median-joining networks in the field of evolutionary biology. Our MJN network revealed the presence of the *er1* gene in the great majority of lines that shared an identical haplotype with the reference PSMLO1 gene, thereby suggesting that these lines have originated from a common ancestor.

## Materials and methods

All the materials collected and the methodology designed for the research was in accordance with relevant guidelines and regulations.

### Collection and identification of test pathogen

A total of 24 isolates of pathogen-causing powdery mildew were collected from North-west Himalayas out of which maximum isolates were collected from 15 different locations in the trans-Himalayan Lahul Spiti region. These were purified and maintained in a greenhouse for further studies. The pathogen causing pea powdery mildew was identified on the basis of morphological characteristics viz., hyphae, conidia, conidiophores, conidia size and conidiophore foot cells. Further, polyphasic analysis of strain was done Internal Transcribed Spacer (ITS) region of nuclear ribosomal DNA (rDNA). The sequence obtained was submitted to the NCBI gene bank for accession number.

### Screening for resistance

Screening for resistance against identified fungal pathogen was done from a panel of 310 pea lines comprising exotic and indigenous germplasm collected from different sources (CSK HPKV Palampur, NBPGR New Delhi, PAU Ludhiana and IIPR Kanpur) were evaluated in net-house as well as on detached leaves under in vitro conditions^54^. The identified resistant lines along with susceptible ones were crossed with known recessive genes *er1* and *er2* present in JI-2302 (*er1*) and JI-2480 (*er2*) lines under greenhouse. Further, cultivars having resistance to respective *er* genes were selected to determine the allelic diversity at *er* locus.

### RNA extraction

A total of 50 pea lines were selected for RNA isolation using the trizol method^55^. RNA was extracted from fresh leaves (without inoculation of *Erysiphe pisi*) and inoculated leaves after 4 and 8 days of fungal (*Erysiphe pisi*) inoculation.

### cDNA Synthesis and amplification studies

40 ug of RNA was used for cDNA amplification using reverse transcriptase enhancer, 5 × cDNA buffer, dNTP mix (5 Mm each), and verso enzyme as per the instructions recommended on the Verso enzyme cDNA kit. PCR reaction mix was incubated at 42 °C for 30 min. Further, the reaction was terminated at 95 °C for 2 min. For amplification of cDNA, PCR plates were filled with a reaction mixture containing 5Xbuffer, 25 mM MgCl2, 10 mM dNTPs, 0.5 mM of each specific-designed PsMLO primer (Table 6), 5U Taq DNA polymerase with template cDNA. Amplification profile consisted of 1 cycle at 95 °C/5 min; 37cycles at 95 °C/30 s, 50 °C/the 30 s and 72 °C/1 min 20 s; 1 cycle at 72 °C/7 min; hold at 4 °C/∞. The PCR products were separated on agarose gel (1.2%) and the targeted amplicons were purified and sequenced at the SciGenome Labs Private Ltd. Cochin, Kerala—INDIA.

**Table 6:** List of primers used for cDNA amplification of pea lines.

Primer name	Sequence (5′–3′)
PsMLO 1 FP	ATGGCTGAAGAGGGAGTTAAGGA
PsMLO1RP	CTAATTGCTCCCTAAGTGGCG CT
PsMLO2FP	CCTCGGAGAATTCTTGCTAC
PsMLO2RP	TCCACAAATCAAGCTGCTACC
PsMLO3FP	TCTGGCTCTTCACAGTGCTT
PsMLO3RP	TGTGGAAGCAAGAGGTTATGG
PsMLOEx5FP	ATGAGGAAGTGGAAGACTTGGGA
PsML015ExRP	GCTTTTTGGCTGTGTGGTGCCAG

### In silico analysis of gene sequences

The homology of gene sequences was analyzed using online bioinformatics tools available in the NCBI database in the FASTA program. BLASTN was used for sequence comparison on NCBI genomic database (http://www.ncbi.nlm.nih.gov/blast/Blast.cgi). Phylogenetic analysis was conducted in MEGA 5.0^56^ and genetic parameters such as haplotype diversity and total number of mutations, Indel polymorphism were calculated using DnaSP version 5.10^57^. Network v 4.61 was used to construct a Median-joining (MJ)^58^ network of the haplotypes (http://www.fluxus-engineering.com).

## Conclusion

For the management of fungal diseases in crops, many strategies including conventional as well as non-conventional approaches are frequently used. From our research, we identified the resistant cultivars in pea crops that meet the demand of low and marginal farmers and reduce the use of chemicals in a controlled manner.

## Supplementary Information

Supplementary Information.

## Data Availability

The datasets analyzed during the current study are available in the NCBI Nucleotide repository, https://www.ncbi.nlm.nih.gov/nuccore/1131300078, https://www.ncbi.nlm.nih.gov/nuccore/1131300079 with accession numbers GenBank: KX455922.1 and GenBank: KX455923.1 respectively.
