# Author Correction: Deciphering diversity at *er* loci for diversification of powdery mildew resistance in pea

**DOI:** 10.1038/s41598-022-25312-0

**Published:** 2022-12-06

**Authors:** Devinder K. Banyal, Himisha Dixit, Jaya Chaudhary, Anudeep B. Malannavar, Nisha Thakur

**Affiliations:** 1 Department of Plant Pathology, COA, CSKHPKV, Palampur, HP 176061 India; 2 Dr YSPUHF, KVK, Chamba, HP 17512 India

Correction to: *Scientific Reports* 10.1038/s41598-022-19894-y, published online 26 September 2022

In the original version of this Article, Himisha Dixit was incorrectly affiliated with ‘Centre for Computational Biology and Bioinformatics, School of Life Sciences, Central University of Himachal Pradesh, TAB Shahpur, Kangra, HP, 176206, India’.

The correct affiliation is listed below.

Department of Plant Pathology, COA, CSKHPKV, Palampur, HP, 176061, India

The original Article has been corrected.

