# Supplementary material for: Deciphering diversity at er loci for diversification of powdery mildew resistance in pea
Source: Sci Rep. 2022 Sep 26;12:16037. doi: 10.1038/s41598-022-19894-y (PMC9512827; doi:10.1038/s41598-022-19894-y)
Supplement: Supplementary file 1 — Supplementary Information. [file 41598_2022_19894_MOESM1_ESM.pdf]

Supplementary information

The datasets analysed during the current study are available in the NCBI Nucleotide repository, https://www.ncbi.nlm.nih.gov/nuccore/1131300078, https://www.ncbi.nlm.nih.gov/nuccore/1131300079 with accession numbers GenBank: KX455922.1 and GenBank: KX455923.1 respectively.

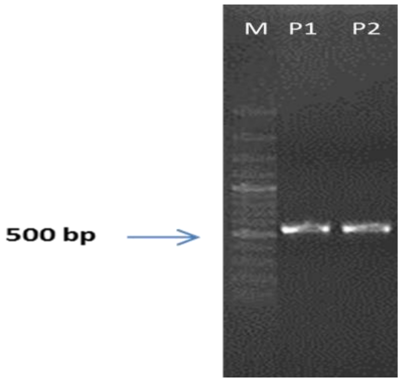

Fig.2 rDNA region amplified using *Erysiphe* specific primers

Multiple sequence alignment of P-1 with *Erysiphe pisi*

P1 with E. pisi

FJ378872.1 ---------------------TCCGTAGGTGAACCTGCGGAAGGATCATTACAGAGTGCG

FJ378868.1 --

FJ378869.1 ---------------------TCCGTAGGTGAACCTGCGGAAGGATCATTACAGAGTGCG

FJ378867.1 ---------------------TCCGTAGGTGAACCTGCGGAAGGATCATTACAGAGTGCG

KP941751.1 --CATTACAGAGTGCG

KP941750.1 --CATTACAGAGTGCG

KF946096.1 ---TACAGAGTGCG

AF011306.1 AAGTAAAAGTCGTAACAAGGTTTCCGTAGGTGAACCTGCGGAAGGATCATACAGAGTGCG

P-1 ---TACAGAGTGCG

KR957355.1 -------GTCGTAACAAGGTTTCCGTAGGTGAACCTGCGGAAGGATCATTACAGAGTGCG

KR912079.1 ------------AACAAGGTTTCCGTAGGTGAACCTGCGGAAGGATCATTACAGAGTGCG

FJ378872.1 GGGCTCAGTCGTGGCATCTGCTGCGTGCTGGGCCGACCCTCCCACCCGTGTCGATTTGTA

FJ378868.1 ----TCAGTCGTGGC-TCTGCTGCGTGCTGGGCCGACCCTCCCACCCGTGTCGATTTGTA

FJ378869.1 AGGCTCAGTCGTGGCATCTGCTGCGTGCTGGGCCGACCCTCCCACCCGTGTCGATTTGTA

FJ378867.1 AGGCTCAGTCGTGGCATCTGCTGCGTGCTGGGCCGACCCTCCCACCCGTGTCGATTTGTA

KP941751.1 AGGCTCAGTCGTGGCATCTGCTGCGTGCTGGGCCGACCCTCCCACCCGTGTCGATTTGTA

KP941750.1 AGGCTCAGTCGTGGCATCTGCTGCGTGCTGGGCCGACCCTCCCACCCGTGTCGATTTGTA

KF946096.1 AGGCTCAGTCGTGGCATCTGCTGCGTGCTGGGCCGACCCTCCCACCCGTGTCGATTTGTA

AF011306.1 AGGCTCAGTCGTGGCATCTGCTGCGTGCTGGGCCGACCCTCCCACCCGTGTCGATTTGTA

P-1 AGGCTCAGTCGTGGCATCTGCTGCGTGCTGGGCCGACCCTCCCACCCGTGTCGATTTGTA

KR957355.1 AGGCTCAGTCGTGGCATCTGCTGCGTGCTGGGCCGACCCTCCCACCCGTGTCGATTTGTA

KR912079.1 AGGCTCAGTCGTGGCATCTGCTGCGTGCTGGGCCGACCCTCCCACCCGTGTCGATTTGTA

*********** **

FJ378872.1 TCTTGTTGCTTTGGCGGGCCGGGCCGCGCTGTCGCTGTCCGCATGGACATGCGTCGGCCG

FJ378868.1 TCTTGTTGCTTTGGCGGGCCGGGCCGCGCTGTCGCTGTCCGCATGGACATGCGTCGGCCG

FJ378869.1 TCTTGTTGCTTTGGCGGGCCGGGCCGCGCTGTCGCTGTCCGCATGGACATGCGTCGGCCG

FJ378867.1 TCTTGTTGCTTTGGCGGGCCGGGCCGCGCTGTCGCTGTCCGCATGGACATGCGTCGGCCG

KP941751.1 TCTTGTTGCTTTGGCGGGCCGGGCCGCGTCGTCGCTGCCTACATGGACATGCGTCGGCCG

KP941750.1 TCTTGTTGCTTTGGCGGGCCGGGCCGCGTCGTCGCTGCCTACATGGACATGCGTCGGCCG

KF946096.1 TCTTGTTGCTTTGGCGGGCCGGGCCGCGCTGTCGCTGTCCGCATGGACATGCGTCGGCCG

AF011306.1 TCTTGTTGCTTTGGCGGGCCGGGCCGCGCTGTCGCTGTCCGCATGGACATGCGTCGGCCG

P-1 TCTTGTTGCTTTGGCGGGCCGGGCCGCGCTGTCGCTGTCCGCATGGACATGCGTCGGCCG

KR957355.1 TCTTGTTGCTTTGGCGGGCCGGGCCGCGCTGTCGCTGTCCGCATGGACATGCGTCGGCCG

KR912079.1 TCTTGTTGCTTTGGCGGGCCGGGCCGCGCTGTCGCTGTCCGCATGGACATGCGTCGGCCG

**************************** ******* * *******************

FJ378872.1 CCCACCGGTTTTCAACTGGAGCGCGCCCGCCAAAGACCCAACCAAAACTCATGTTGTTTG

FJ378868.1 CCCACCGGTTTTCAACTGGAGCGCGCCCGCCAAAGACCCAACCAAAACTCATGTTGTTTG

FJ378869.1 CCCACCGGTTTTCAACTGGAGCGCGCCCGCCAAAGACCCAACCAAAACTCATGTTGTTTG

FJ378867.1 CCCACCGGTTTTCAACTGGAGCGCGCCCGCCAAAGACCCAACCAAAACTCATGTTGTTTG

KP941751.1 CCCACCGGTTTTCAACTGGAGCGCGCCCGCCAAAGACCCAACCAAAACTCATGTTGTTTG

KP941750.1 CCCACCGGTTTTCAACTGGAGCGCGCCCGCCAAAGACCCAACCAAAACTCATGTTGTTTG

KF946096.1 CCCACCGGTTTTCAACTGGAGCGCGCCCGCCAAAGACCCAACCAAAACTCATGTTGTTTG

AF011306.1 CCCACCGGTTTTCAACTGGAGCGCGCCCGCCAAAGACCCAACCAAAACTCATGTTGTTTG

P-1 CCCACCGGTTTTCAACTGGAGCGCGCCCGCCAAAGACCCAACCAAAACTCATGTTGTTTG

KR957355.1 CCCACCGGTTTTCAACTGGAGCGCGCCCGCCAAAGACCCAACCAAAACTCATGTTGTTTG

KR912079.1 CCCACCGGTTTTCAACTGGAGCGCGCCCGCCAAAGACCCAACCAAAACTCATGTTGTTTG

****

FJ378872.1 TGTCGTCTCAGCTTTATTATGAAAATTGATAAAACTTTCAACAACGGATCTCTTGGCTCT

FJ378868.1 TGTCGTCTCAGCTTTATTATGAAAATTGATAAAACTTTCAACAACGGATCTCTTGGCTCT

FJ378869.1 TGTCGTCTCAGCTTTATTATGAAAATTGATAAAACTTTCAACAACGGATCTCTTGGCTCT

FJ378867.1 TGTCGTCTCAGCTTTATTATGAAAATTGATAAAACTTTCAACAACGGATCTCTTGGCTCT

KP941751.1 TGTCGTCTCAGCTATATTATGAAAATTGATAAAACTTTCAACAACGGATCTCTTGGCTCT

KP941750.1 TGTCGTCTCAGCTATATTATGAAAATTGATAAAACTTTCAACAACGGATCTCTTGGCTCT

KF946096.1 TGTCGTCTCAGCTTTATTATGAAAATTGATAAAACTTTCAACAACGGATCTCTTGGCTCT

AF011306.1 TGTCGTCTCAGCTTTATTATGAAAATTGATAAAACTTTCAACAACGGATCTCTTGGCTCT

P-1 TGTCGTCTCAGCTTTATTATGAAAATTGATAAAACTTTCAACAACGGATCTCTTGGCTCT

KR957355.1 TGTCGTCTCAGCTTTATTATGAAAATTGATAAAACTTTCAACAACGGATCTCTTGGCTCT

KR912079.1 TGTCGTCTCAGCTTTATTATGAAAATTGATAAAACTTTCAACAACGGATCTCTTGGCTCT

************* **

FJ378872.1 GGCATCGATGAAGAACGCAGCGAAATGCGATAAGTAATGTGAATTGCAGAATTTAGTGAA

FJ378868.1 GGCATCGATGAAGAACGCAGCGAAATGCGATAAGTAATGTGAATTGCAGAATTTAGTGAA

FJ378869.1 GGCATCGATGAAGAACGCAGCGAAATGCGATAAGTAATGTGAATTGCAGAATTTAGTGAA

FJ378867.1 GGCATCGATGAAGAACGCAGCGAAATGCGATAAGTAATGTGAATTGCAGAATTTAGTGAA

KP941751.1 GGCATCGATGAAGAACGCAGCGAAATGCGATAAGTAATGTGAATTGCAGAATTTAGTGAA

KP941750.1 GGCATCGATGAAGAACGCAGCGAAATGCGATAAGTAATGTGAATTGCAGAATTTAGTGAA

KF946096.1 GGCATCGATGAAGAACGCAGCGAAATGCGATAAGTAATGTGAATTGCAGAATTTAGTGAA

AF011306.1 GGCATCGATGAAGAACGCAGCGAAATGCGATAAGTAATGTGAATTGCAGAATTTAGTGAA

P-1 GGCATCGATGAAGAACGCAGCGAAATGCGATAAGTAATGTGAATTGCAGAATTTAGTGAA

KR957355.1 GGCATCGATGAAGAACGCAGCGAAATGCGATAAGTAATGTGAATTGCAGAATTTAGTGAA

KR912079.1 GGCATCGATGAAGAACGCAGCGAAATGCGATAAGTAATGTGAATTGCAGAATTTAGTGAA

****

FJ378872.1 TCATCGAATCTTTGAACGCACATTGCGCCCCTTGGTATTCCGAGGGGCATGCCTGTTCGA

FJ378868.1 TCATCGAATCTTTGAACGCACATTGCGCCCCTTGGTATTCCGAGGGGCATGCCTGTTCGA

FJ378869.1 TCATCGAATCTTTGAACGCACATTGCGCCCCTTGGTATTCCGAGGGGCATGCCTGTTCGA

FJ378867.1 TCATCGAATCTTTGAACGCACATTGCGCCCCTTGGTATTCCGAGGGGCATGCCTGTTCGA

KP941751.1 TCATCGAATCTTTGAACGCACATTGCGCCCCTTGGTATTCCGAGGGGCATGCCTGTTCGA

KP941750.1 TCATCGAATCTTTGAACGCACATTGCGCCCCTTGGTATTCCGAGGGGCATGCCTGTTCGA

KF946096.1 TCATCGAATCTTTGAACGCACATTGCGCCCCTTGGTATTCCGAGGGGCATGCCTGTTCGA

AF011306.1 TCATCGAATCTTTGAACGCACATTGCGCCCCTTGGTATTCCGAGGGGCATGCCTGTTCGA

P-1 TCATCGAATCTTTGAACGCACATTGCGCCCCTTGGTATTCCGAGGGGCATGCCTGTTCGA

KR957355.1 TCATCGAATCTTTGAACGCACATTGCGCCCCTTGGTATTCCGAGGGGCATGCCTGTTCGA

KR912079.1 TCATCGAATCTTTGAACGCACATTGCGCCCCTTGGTATTCCGAGGGGCATGCCTGTTCGA

****

FJ378872.1 GCGTCATAACACCCCCTCCAGCTGCCTTTGTGTGGCTGCGGTGTTGGGGCTCGTCGCGAA

FJ378868.1 GCGTCATAACACCCCCTCCAGCTGCCTTTGTGTGGCTGCGGTGTTGGGGCTCGTCGCGAA

FJ378869.1 GCGTCATA-CACCCCCTCCAGCTGCCTTTGTGTGGCTGCGGTGTTGGGGCTCGTCGCGAA

FJ378867.1 GCGTCATAACACCCCCTCCAGCTGCCTTTGTGTGGCTGCGGTGTTGGGGCTCGTCGCGAA

KP941751.1 GCGTCATAACACCCCCTCCAGCTGCCTTTGTGTGGCTGCGGTGTTGGGGCTCGTCGCGAT

KP941750.1 GCGTCATAACACCCCCTCCAGCTGCCTTTGTGTGGCTGCGGTGTTGGGGCTCGTCGCGAT

KF946096.1 GCGTCATAACACCCCCTCCAGCTGCCTTTGTGTGGCTGCGGTGTTGGGGCTCGTCGCGAA

AF011306.1 GCGTCATAACACCCCCTCCAGCTGCCTTTGTGTGGCTGCGGTGTTGGGGCTCGTCGCGAA

P-1 GCGTCATAACACCCCCTCCAGCTGCCTTTGTGTGGCTGCGGTGTTGGGGCTCGTCGCGAA

KR957355.1 GCGTCATAACACCCCCTCCAGCTGCCTTTGTGTGGCTGCGGTGTTGGGGCTCGTCGCGAA

KR912079.1 GCGTCATAACACCCCCTCCAGCTGCCTTTGTGTGGCTGCGGTGTTGGGGCTCGTCGCGAA

******** ***

FJ378872.1 GCGGCGGCCCTTAAAGACAGTGGCGGTCCCGGCGTGGGCTCTACGCGTAGTAACTTGCTT

FJ378868.1 GCGGCGGCCCTTAAAGACAGTGGCGGTCCCGGCGTGGGCTCTACGCGTAGTAACTTGCTT

FJ378869.1 GCGGCGGCCCTTAAAGACAGTGGCGGTCCCGGCGTGGGCTCTACGCGTAGTAACTTGCTT

FJ378867.1 GCGGCGGCCCTTAAAGACAGTGGCGGTCCCGGCGTGGGCTCTACGCGTAGTAACTTGCTT

KP941751.1 GCGGCGGCCCTTAAAGACAGTGGCGGTCCCGGCGTGGGCTCTACGCGTAGTAACTTGCTT

KP941750.1 GCGGCGGCCCTTAAAGACAGTGGCGGTCCCGGCGTGGGCTCTACGCGTAGTAACTTGCTT

KF946096.1 GCGGCGGCCCTTAAAGACAGTGGCGGTCCCGGCGTGGGCTCTACGCGTAGTAACTTGCTT

AF011306.1 GCGGCGGCCCTTAAAGACAGTGGCGGTCCCGGCGTGGGCTCTACGCGTAGTAACTTGCTT

P-1 GCGGCGGCCCTTAAAGACAGTGGCGGTCCCGGCGTGG-CTCTACGCGTAGTAACTTGCTT

KR957355.1 GCGGCGGCCCTTAAAGACAGTGGCGGTCCCGGCGTGGGCTCTACGCGTAGTAACTTGCTT

KR912079.1 GCGGCGGCCCTTAAAGACAGTGGCGGTCCCGGCGTGGGCTCTACGCGTAGTAACTTGCTT

************************************* **********************

FJ378872.1 CTCGCGACAGAGTGACGACGGTGGCTTGCCAGAACAACCCTCATCTGCTCCAGTCACATG

FJ378868.1 CTCGCGACAGAGTGACGACGGTGGCTTGCCAGAACAACCCTCATCTGCTCCAGTCACATG

FJ378869.1 CTCGCGACAGAGTGACGACGGTGGCTTGCCAGAACAACCCTCATCTGCTCCAGTCACATG

FJ378867.1 CTCGCGACAGAGTGACGACGGTGGCTTGCCAGAACAACCCTCATCTGCTCCAGTCACATG

KP941751.1 CTCGCGACAGAGTGGCGACGGTGGCTTGCCAAAACAACCCTTTTCTGCTCCAGTCATATG

KP941750.1 CTCGCGACAGAGTGGCGACGGTGGCTTGCCAAAACAACCCTTTTCTGCTCCAGTCATATG

KF946096.1 CTCGCGACAGAGTGACGACGGTGGCTTGCCAGAACAACCCTCATCTGCTCCAGTCACATG

AF011306.1 CTCGCGACAGAGTGACGACGGTGGCTTGCCAGAACAACCCTCATCTGCTCCAGTCACATG

P-1 CTCGCGACAGAGTGACGACG-TGGCTTGCCAGAACAACCCTCATCTGCTCCAGTCACATG

KR957355.1 CTCGCGACAGAGTGACGACGGTGGCTTGCCAGAACAACCCTCATCTGCTCCAGTCACATG

KR912079.1 CTCGCGACAGAGTGACGACGGTGGCTTGCCAGAACAACCCTCATCTGCTCCAGTCACATG

************** ***** ********** ********* ************* ***

FJ378872.1 GATCACAGGTTGACCTCGAATCAGGTAG--GAATA----CCCGCTGAACTTAAGCATATC

FJ378868.1 GATCACAGGTTGACCTCGAATCAGGTAG--GAATA----CCCGCTGAACTTAAGCATATC

FJ378869.1 GATCACAGGTTGACCTCGAATCAGGTAG--GAATA----CCCGCTGAACTTAAGCATATC

FJ378867.1 GATCACAGGTTGACCTCGAATCAGGTAG--GAATA----CCCGCTGAACTTAAGCATATC

KP941751.1 GATCACAGGTTGACCTCGAATCAGGTAG--GAATA----CCCGCTGAACTTAAGCATATC

KP941750.1 GATCACAGGTTGACCTCGAATCAGGTAG--GAATA----CCCGCTGAACTTAAGCATATC

KF946096.1 GATCACAGGTTGACCTCGAATCAGGTAG--GAATA----CCCGCTGAACTTAAGCATATC

AF011306.1 GATCACAGGTTGACCTCGAATCAGGTAG--GAATA----CCCGCTGAACTTAAGCATATC

P-1 GATCACAGGTTGACC---

KR957355.1 GATCACAGGTTGACCTCGAATCAGGTAGATGAATCTTATCCCGCTGAACTTAAGCATATC

KR912079.1 GATCACAGGTTGACCTCGAATCAGGTAG--GAATA----CCCGCTGAACTTAAGCATATC

****

Multiple sequence alignment of P-2 with *Erysiphe trifolii*

P2 with E. trifolii

GU361633.1 --
GU361634.1 --
FJ378884.1 --TACAGAGTGCGAGGCTCAGT
AB591735.1 -------------CCGTAGGTGAACCTGCGGAAGGATCATTACAGAGTGCGAGGCTCAGT
AB104521.1 --CAGAGTGCGAGGCTCAGT
FJ378878.1 ------------TCCGTAGGTGAACCTGCGGAAGGATCATTACAGAGTGCGAGGCTCAGT
FJ378873.1 ------------TCCGTAGGTGAACCTGCGGAAGGATCATTACAGAGTGCGAGGCTCAGT
FJ378874.1 ------------TCCGTAGGTGAACCTGCGGAAGGATCATTACAGAGTGCGAGGCTCAGT
FJ378881.1 --TACAGAGTGCGAGGCTCAGT
FJ378882.1 --TACAGAGTGCGAGGCTCAGT
FJ378877.1 --------------------------TGCGGAAGGATCATTACAGAGTGCGAGGCTCAGT
JQ282732.1 CGTAACAAGGTTTCCGTAGGTGAACCTGCGGAAGGATCATTACAGAGTGCGAGGCTCAGT
P-2 --TACAGAGTGCGAGGCTCAGT
GU361633.1 -------TCTGCCGCGTGCTGGGCCGACCCTCCCACCCGTGTCGATTTGTATCTTGTTGC
GU361634.1 -------TCTGCCGCGTGCTGGGCCGACCCTCCCACCCGTGTCGATTTGTATCTTGTTGC
FJ378884.1 CGCGGCGTCTGCCGCGTGCTGGGCCGACCCTCCCACCCGTGTCGATTTGTATCTTGTTGC
AB591735.1 CGCGGCGTCGGCCGCGTGCTGGGCCGACCCTCCCACCCGTGTCGATTTGTATCTTGTTGC
AB104521.1 CGCGGCGTCTGCCGCGTGCTGGGCCGACCCTCCCACCCGTGTCGATTTGTATCTTGTTGC
FJ378878.1 CGCGGCGTCTGCCGCGTGCTGGGCCGACCCTCCCACCCGTGTCGATTTGTATCTTGTTGC
FJ378873.1 CGCGGCGTCTGCC-CGTGCTGGGCCGACCCTCCCACCCGTGTCGATTTGTATCTTGTTGC
FJ378874.1 CGCGGCGTCTGCCGCGTGCTGGGCCGACCCTCCCACCCGTGTCGATTTGTATCTTGTTGC
FJ378881.1 CGCGGCGTCTGCCGCGTGCTGGGCCGACCCTCCCACCCGTGTCGATTTGTATCTTGTTGC
FJ378882.1 CGCGGCGTCTGCCGCGTGCTGGGCCGACCCTCCCACCCGTGTCGATTTGTATCTTGTTGC
FJ378877.1 CGCGGCGTCTGCCGCGTGCTGGGCCGACCCTCCCACCCGTGTCGATTTGTATCTTGTTGC
JQ282732.1 CGCGGCGTCTGCCGCGTGCTGGGCCGACCCTACCACCCGTGTCGATTTGTATCTTGTTGC
P-2 CGCGGCGTCTGCCGCGTGCTGGGCCGACC-TCCCACCCGTGTCGATTTGTATCTTGTTGC
** *** *************** * ****************************
GU361633.1 TTTGGCGGGCCGGGCCGCGTCGTCGCTGTTCGCAAGGACCTGCGTCGGCCGCCCACCGGT
GU361634.1 TTTGGCGGGCCGGGCCGCGTCGTCGCTGTTCGCAAGGACCTGCGTCGGCCGCCCACCGGT
FJ378884.1 TTTGGCGGGCCGGGCCGCGTCGTCGCTGTTCGCAAGGACCTGCGTCGGCCGCCCACCGGT
AB591735.1 TTTGGCGGGCCGGGCCGCGTCGTCGCTGTTCGCAAGGACGTGCGTCGGCCGCCCACCGGT
AB104521.1 TTTGGCGGGCCGGGCCGCGTCGTCGCTGTCCGCAAGGACCTGCGTCGGCCGCCCACCGGT
FJ378878.1 TTTGGCGGGCCGGGCCGCGTCGTCGCTGTTCGCAAGGACCTGCGTCGGCCGCCCACCGGT
FJ378873.1 TTTGGCGGGCCGGGCCGCGTCGTCGCTGTTCGCAAGGACCTGCGTCGGCCGCCCACCGGT
FJ378874.1 TTTGGCGGGCCGGGCCGCGTCGTCGCTGTTCGCAAGGACCTGCGTCGGCCGCCCACCGGT
FJ378881.1 TTTGGCGGGCCGGGCCGCGTCGTCGCTGTTCGCAAGGACCTGCGTCGGCCGCCCACCGGT
FJ378882.1 TTTGGCGGGCCGGGCCGCGTCGTCGCTGTTCGCAAGGACCTGCGTCGGCCGCCCACCGGT
FJ378877.1 TTTGGCGGGCCGGGCCGCGTCGTCGCTGTTCGCAAGGACCTGCGTCGGCCGCCCACCGGT
JQ282732.1 TTTGGCGGGCCGGGCCGCGTCGTCGCTGTTCGCAAGGACCTGCGTCGGCCGCCCACCGGT
P-2 TT-GGCGGGCCGGGCCG-GTCGTCGCTGTTCGCAAGGACCTGCGTCGGCCGCCCACCGGT
** ************** *********** ********* *********************
GU361633.1 TTTGAACTGGAGCGCGCCCGCCAAAGACCCAACCAAAACTCATGTTGTTTGTGTCGTCTC
GU361634.1 TTTGAACTGGAGCGCGCCCGGCAAAGACCCAACCAAAACTCATGTTGTTTGTGTCGTCTC
FJ378884.1 TTTGAACTGGAGCGCGCCCGCCAAAGACCCAACCAAAACTCATGTTGTTTGTGTCGTCTC
AB591735.1 TTAGAACTGGAGCGCGCCCGCCAAAGACCCAACCAAAACTCATGTTGTTTGTGTCGTCTC
AB104521.1 TTAGAACTGGAGCGCGCCCGCCAAAGACCCAACCAAAACTCATGTTGTTTGTGTCGTCTC
FJ378878.1 TTTGAACTGGAGCGCGCCCGCCAAAGACCCAACCAAAACTCATGTTGTTTGTGTCGTCTC
FJ378873.1 TTTGAACTGGAGCGCGCCCGCCAAAGACCCAACCAAAACT-ATGTTGTTTGTGTCGTCTC
FJ378874.1 TTTGAACTGGAGCGCGCCCGCCAAAGACCCAACCAAAACTCATGTTGTTTGTGTCGTCTC
FJ378881.1 TTTGAACTGGAGCGCGCCCGCCAAAGACCCAACCAAAACTCATGTTGTTTGTGTCGTCTC

FJ378882.1 TTTGAACTGGAGCGCGCCCGCCAAAGACCCAACCAAAACTCATGTTGTTTGTGTCGTCTC
FJ378877.1 TTTGAACTGGAGCGCGCCCGCCAAAGACCCAACCAAAACTCATGTTGTTTGTGTCGTCTC
JQ282732.1 TTTGAACGGGAGCGCGCCCGCCAAAGACCCAACCAAAACTCATGTTGTTTGTGTCGTCTC
P-2 TTTGAACTGGAGCGCGCCCGCCAAAGACCCAACCAAAACTCATGTTGTTTGTGTCGTCTC
** **** ************ ******************* *******************

GU361633.1 AGCTTTATTATGAAAATTGATAAAACTTTCAACAACGGATCTCTTGGCTCTGGCATCGAT
GU361634.1 AGCTTTATTATGAAAATTGATAAAACTTTCAACAACGGATCTCTTGGCTCTGGCATCGAT
FJ378884.1 AGCTTTATTATGAAAATTGATAAAACTTTCAACAACGGATCTCTTGGCTCTGGCATCGAT
AB591735.1 AGCTTTATTATGAAAATTGATAAAACTTTCAACAACGGATCTCTTGGCTCTGGCATCGAT
AB104521.1 AGCTTTATTATGAAAATTGATAAAACTTTCAACAACGGATCTCTTGGCTCTGGCATCGAT
FJ378878.1 AGCTTTATTATGAAAATTGATAAAACTTTCAACAACGGATCTCTTGGCTCTGGCATCGAT
FJ378873.1 AGCTTTATTATGAAAATTGATAAAACTTTCAACAACGGATCTCTTGGCTCTGGCATCGAT
FJ378874.1 AGCTTTATTATGAAAATTGATAAAACTTTCAACAACGGATCTCTTGGCTCTGGCATCGAT
FJ378881.1 AGCTTTATTATGAAAATTGATAAAACTTTCAACAACGGATCTCTTGGCTCTGGCATCGAT
FJ378882.1 AGCTTTATTATGAAAATTGATAAAACTTTCAACAACGGATCTCTTGGCTCTGGCATCGAT
FJ378877.1 AGCTTTATTATGAAAATTGATAAAACTTTCAACAACGGATCTCTTGGCTCTGGCATCGAT
JQ282732.1 AGCTTTATTATGAAAATTGATAAAACTTTCAACAACGGATCTCTTGGCTCTGGCATCGAT
P-2 AGCTTTATTATGAAAATTGATAAAACTTTCAACAACGGATCTCTTGGCTCTGGCATCGAT
****

GU361633.1 GAAGAACGCAGCGAAATGCGATAAGTAATGTGAATTGCAGAATTTAGTGAATCATCGAAT
GU361634.1 GAAGAACGCAGCGAAATGCGATAAGTAATGTGAATTGCAGAATTTAGTGAATCATCGAAT
FJ378884.1 GAAGAACGCAGCGAAATGCGATAAGTAATGTGAATTGCAGAATTTAGTGAATCATCGAAT
AB591735.1 GAAGAACGCAGCGAAATGCGATAAGTAATGTGAATTGCAGAATTTAGTGAATCATCGAAT
AB104521.1 GAAGAACGCAGCGAAATGCGATAAGTAATGTGAATTGCAGAATTTAGTGAATCATCGAAT
FJ378878.1 GAAGAACGCAGCGAAATGCGATAAGTAATGTGAATTGCAGAATTTAGTGAATCATCGAAT
FJ378873.1 GAAGAACGCAGCGAAATGCGATAAGTAATGTGAATTGCAGAATTTAGTGAATCATCGAAT
FJ378874.1 GAAGAACGCAGCGAAATGCGATAAGTAATGTGAATTGCAGAATTTAGTGAATCATCGAAT
FJ378881.1 GAAGAACGCAGCGAAATGCGATAAGTAATGTGAATTGCAGAATTTAGTGAATCATCGAAT
FJ378882.1 GAAGAACGCAGCGAAATGCGATAAGTAATGTGAATTGCAGAATTTAGTGAATCATCGAAT
FJ378877.1 GAAGAACGCAGCGAAATGCGATAAGTAATGTGAATTGCAGAATTTAGTGAATCATCGAAT
JQ282732.1 GAAGAACGCAGCGAAATGCGATAAGTAATGTGAATTGCAGAATTTAGTGAATCATCGAAT
P-2 GAAGAACGCAGCGAAATGCGATAAGTAATGTGAATTGCAGAATTTAGTGAATCATCGAAT
****

GU361633.1 CTTTGAACGCACATTGCGCCCCTTGGTATTCCGAGGGGCATGCCTGTTCGAGCGTCATAA
GU361634.1 CTTTGAACGCACATTGCGCCCCTTGGTATTCCGAGGGGCATGCCTGTTCGAGCGTCATAA
FJ378884.1 CTTTGAACGCACATTGCGCCCCTTGGTATTCCGAGGGGCATGCCTGTTCGAGCGTCATAA
AB591735.1 CTTTGAACGCACATTGCGCCCCTTGGTATTCCGAGGGGCATGCCTGTTCGAGCGTCATAA
AB104521.1 CTTTGAACGCACATTGCGCCCCTTGGTATTCCGAGGGGCATGCCTGTTCGAGCGTCATAA
FJ378878.1 CTTTGAACGCACATTGCGCCCCTTGGTATTCCGAGGGGCATGCCTGTTCGAGCGTCATAA
FJ378873.1 CTTTGAACGCACATTGCGCCCCTTGGTATTCCGAGGGGCATGCCTGTTCGAGCGTCATAA
FJ378874.1 CTTTGAACGCACATTGCGCCCCTTGGTATTCCGAGGGGCATGCCTGTTCGAGCGTCATAA
FJ378881.1 CTTTGAACGCACATTGCGCCCCTTGGTATTCCGAGGGGCATGCCTGTTCGAGCGTCATAA
FJ378882.1 CTTTGAACGCACATTGCGCCCCTTGGTATTCCGAGGGGCATGCCTGTTCGAGCGTCATAA
FJ378877.1 CTTTGAACGCACATTGCGCCCCTTGGTATTCCGAGGGGCATGCCTGTTCGAGCGTCATAA
JQ282732.1 CTTTGAACGCACATTGCGCCCCTTGGTATTCCGAGGGGCATGCCTGTTCGAGCGTCATAA
P-2 CTTTGAACGCACATTGCGCCCCTTGGTATTCCGAGGGGCATGCCTGTTCGAGCGTCATAA
****

GU361633.1 CACCCCCTCCAGCTGCCTTTGTGTGGCTGCGGTGTTGGGGCACGTCTCGATGCGGCGGCC
GU361634.1 CACCCCCTCCAGCTGCCTTTGTGTGGCTGCGGTGTTGGGGCACGTCTCGATGCGGCGGCC
FJ378884.1 CACCCCCTCCAGCTGCCTTTGTGTGGCTGCGGTGTTGGGGCACGTCGCGATGCGGCGGCC
AB591735.1 CACCCCCTCCAGCTGCCTTTGTGTGGCTGCGGTGTTGGGGCACGTCGCGATGCGGCGGCC
AB104521.1 CACCCCCTCCAGCTGCCTTTGTGTGGCTGCGGTGTTGGGGCCCGTCGCGATGCGGCGGCC
FJ378878.1 CACCCCCTCCAGCTGCCTTTGTGTGGCTGCGGTGTTGGGGCACGTCGCGACGCGGCGGCC
FJ378873.1 CACCCCCTCCAGCTGCCTTTGTGTGGCTGCGGTGTTGGGGCACGTCGCGATGCGGCGGCC
FJ378874.1 CACCCCCTCCAGCTGCCTTTGTGTGGCTGCGGTGTTGGGGCACGTCGCGATGCGGCGGCC
FJ378881.1 CACCCCCTCCAGCTGCCTTTGTGTGGCTGCGGTGTTGGGGCACGTCGCGATGCGGCGGCC
FJ378882.1 CACCCCCTCCAGCTGCCTTTGTGTGGCTGCGGTGTTGGGGCACGTCGCGATGCGGCGGCC
FJ378877.1 CACCCCCTCCAGCTGCCTTTGTGTGGCTGCGGTGTTGGGGCACGTCGCGATGCGGCGGCC
JQ282732.1 CACCCCCTCCAGCTGCCTTTGTGTGGCTGCGGTGTTGGGGCACGTCGCGATGCGGCGGCC
P-2 CACCCCCTCCAGCTGCCTTTGTGTGGCTGCGGTGTTGGGGCACGTCGCGATGCGGCGGCC
*** **** *** *********

GU361633.1 CTTAAAGACAGTGGCGGTCCCGGCGTGGGCTCTACTCGTAGTAACTTGCTTCTCGCGACA
GU361634.1 CTTAAAGACAGTGGCGGTCCCGGCGTGGGCTCTACTCGTAGTAACTTGCTTCTCGCGACA
FJ378884.1 CTTAAAGACAGTGGCGGTCCCGGCGTGGGCTCTACGCGTAGTAACTTGCTTCTCGCGACA
AB591735.1 CTTAAAGACAGTGGCGGTCCCGGCGTGGGCTCTACGCGTAGTAACTTGCTTCTCGCGACA
AB104521.1 CTTAAAGACAGTGGCGGTCCCGGCGTGGGCTCTACGCGTAGTAACTTGCTTCTCGCGACA
FJ378878.1 CTTAAAGACAGTGGCGGTCCCGGCGTGGGCTCTACGCGTAGTAACTTGCTTCTCGCGACA
FJ378873.1 CTTAAAGACAGTGGCGGTCCCGGCGTGGGCTCTACGCGTAGTAACTTGCTTCTCGCGACA
FJ378874.1 CTTAAAGACAGTGGCGGTCCCGGCGTGGGCTCTACGCGTAGTAACTTGCTTCTCGCGACA
FJ378881.1 CTTAAAGACAGTGGCGGTCCCGGCGTGGGCTCTACGCGTAGTAACTTGCTTCTCGCGACA
FJ378882.1 CTTAAAGACAGTGGCGGTCCCGGCGTGGGCTCTACGCGTAGTAACTTGCTTCTCGCGACA
FJ378877.1 CTTAAAGACAGTGGCGGTCCCGGCGTGGGCTCTACGCGTAGTAACTTGCTTCTCGCGACA
JQ282732.1 CTTAAAGACAGTGGCGGTCCCGGCGTGGGCTCTACGCGTAGTAACTTGCTTCTCGCGACA
P-2 CTTAAAGACAGTGGCGGTCCCGGCGTGGGCTCTACGCGTAGTAACTTGCTTCTCGCGACA
********************************** *************************

GU361633.1 GACTGACGACGGTGGCTTGCCAGAACACCCCTCTTTTGCTCCAGTCACATGGATCACAGG
GU361634.1 GAGTGACGACGGTGGCTTGCCAGAACAACCCTCTTTTGCTCCAGTCACATGGATCACAGG
FJ378884.1 GAGTGACGACGGTGGCTTGCCAGAACACCCCTCTTTTGCTCCAGTCACATGGATCACAGG
AB591735.1 GAGTGACGACGGTGGCTTGCCAGAACACCCCTCTTTTGCTCCAGTCACATGGATCACAGG

AB104521.1 GAGTGACGACGGTGGCTTGCCAGAACACCCCTCTTTTGCTCCAGTCACATGGATCACAGG
FJ378878.1 GAGTGACGACGGTGGCTTGCCAGAACACCCCTCTTTTGCTCCAGTCACATGGATCACAGG
FJ378873.1 GAGTGACGACGGTGGCTTGCCAGAACACCCCTCTTTTGCTCCAGTCACATGGATCACAGG
FJ378874.1 GAGTGACGACGGTGGCTTGCCAGAACACCCCTCTTTTGCTCCAGTCACATGGATCACAGG
FJ378881.1 GAGTGACGACGGTGGCTTGCCAGAACACCCCTCTTTTGCTCCAGTCACATGGATCACAGG
FJ378882.1 GAGTGACGACGGTGGCTTGCCAGAACACCCCTCTTTTGCTCCAGTCACATGGATCACAGG
FJ378877.1 GAGTGACGACGGTGGCTTGCCAGAACACCCCTCTTTTGCTCCAGTCACATGGATCACAGG
JQ282732.1 GAGTGACGACGGTGGCTTGCCAGAACACCCCTCTTTTGCTCCAGTCACATGGATCACAGG
P-2 GAGTGACGACAGTGGCTTGCCAGAACACCCCTCTGTTGCTCCAGTCACATGGATCACAGG
** ******* **************** ****** ************************
GU361633.1 TTGACCTCGAATCAGG--
GU361634.1 --
FJ378884.1 TTGACCTCGAATCAGGTAGGAATACCCGCTGAACTTAAGCATATCAATAAGCGGAGGA
AB591735.1 TTGACCTCGAATCAGGTAGGAATACCCGCTGAACTTAAGCATATCAATAAGCGGAGGA
AB104521.1 TTGACCTCGAATCAGGTAGGAAT-----------------------------------
FJ378878.1 TTGACCTCGAATCAGGTAGGAATACCCGCTGAACTTAAGCATATCAATAAGCGGAGGA
FJ378873.1 TTGACCTCGAATCAGGTAGGAATACCCGCTGAACTTAAGCATATCAATAAGCGGAGGA
FJ378874.1 TTGACCTCGAATCAGGTAGGAATACCCGCTGAACTTAAGCATATCAATAAGCGGAGGA
FJ378881.1 TTGACC--
FJ378882.1 TTGACCAA--
FJ378877.1 TTGACC--
JQ282732.1 TTGACCTCGAATCAGGTAGGAATACCCGCTGAACTTAAGCATATCA------------
P-2 TTGACC--

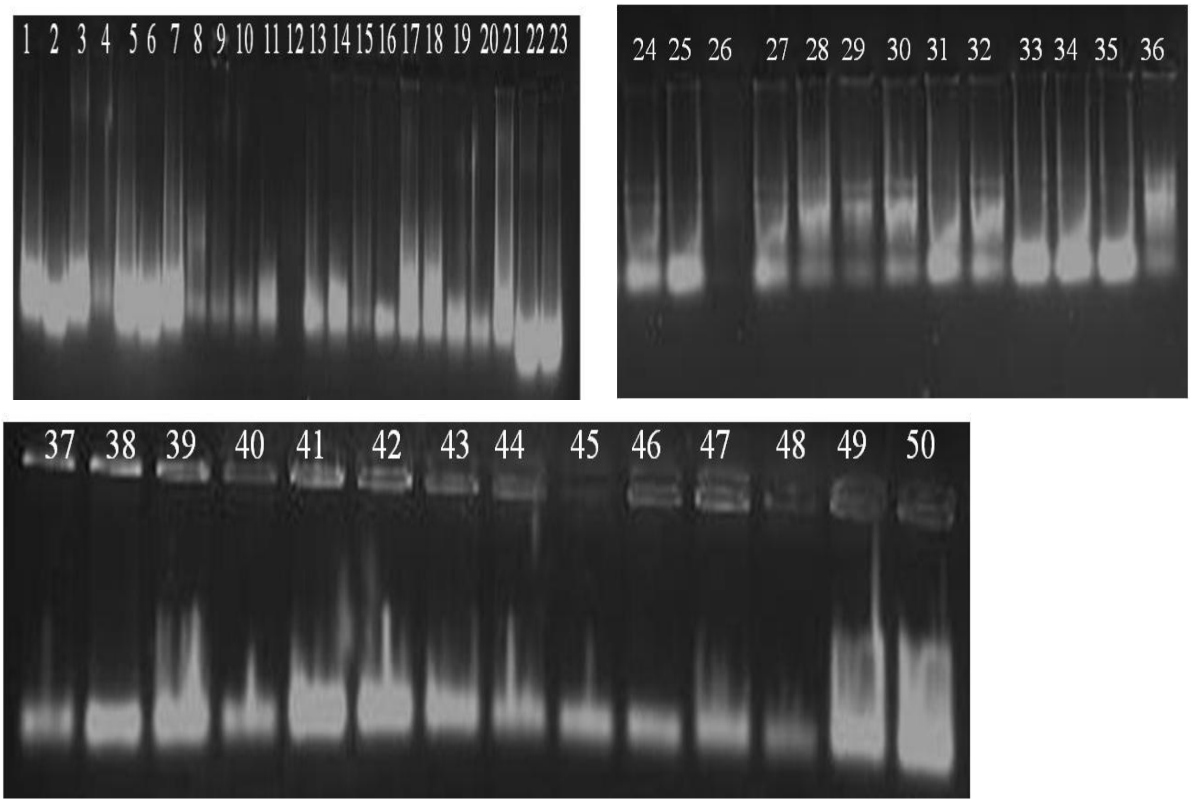

Fig. 3 RNA isolation using Trizol method (1-50 lines)

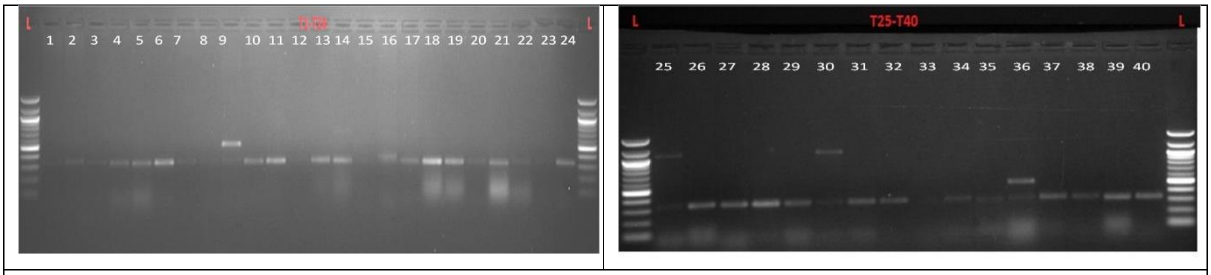

Fig.3 Amplification profile of selected pea lines (1-40) with primer pair PsMLO3F and PsMLO3R

The target amplicons were purified and sequenced at the SciGenom Labs Private Ltd. Cochin, Kerala - INDIA. Primer PsMLO3F and PsMLO3R produced 40 amplicons of variable size (300-325 bp) in different genotypes used. Gel images of PCR product was photographed at SciGenom Labs Private Ltd. Cochin, Kerala - INDIA.

FASTA sequences of 36 haplotypes

>FJ463618.1
AAAACAACAAGTCAAAAAAGAAAGAAAAAATGGCTGAAGAGGGAGTTAAGGAACGAACTTTGGAAGAAACACCAACTTGGGC
TGTTGCAGTTGTGTGTCTTGTGTTGCTAGCTGTTTCAATCTTAATTGAACATATTATTCATGTTATTGGAAAGTGGTTGAAGAAGA
GAAACAAAAATGCTCTTTATGAAGCTTTGGAAAAGATCAAAGGAGAGCTTATGCTACTAGGATTCATATCCTTGCTTCTAACTGT
CTTCCAAGATAATATTTCTAAAATATGCGTATCACAAAAAATTGGATCAACTTGGCATCCTTGTTCCACTTCAAACACAAAGGCCA
AGGCTAAATCTGATGAATCATTAGACTATAAAACCAACAATGATAGAAAACTCTTGGAGTATTTTGATCCTATTCCTCGGAGAATT
CTTGCTACAAAAGGATATGATAAATGTTTTGATAGGGTCAAGTTGCATTAGTTTCTGCATATGGAATTCACCAACTCCATATATTC
ATTTTTGTGCTGGCACTATTTCATATCCTTCAATGTATAATAACATTAACTTTGGGAAGAATCAAGATGAGGAAGTGGAAGACTT
GGGAAGATGAGACAAGAACAGTTGAATATCAATTTTATAATGATCCTGAGAGGTTTAGGTTTGCAAGGGACACAACATTTGGAA
GAAGGCACTTGAGCATGTGGGCTCAGTCACCTATTTTGTTATGGATTGTTAGCTTCTTCAGACAATTCTTTGGATCTATCAGTAGA
GTTGATTATATGGCTCTTAGGCATGGATTTATCATGGCTCATCTTCCTCCAGGACATGATGCACAATTTGATTTCCAAAAGTATAT
AAGTAGATCAATTGAAGAGGATTTTAAAGTTGTTGTAGGAATAAGTCCAACTATCTGGCTCTTCACAGTGCTTTTCCTTCTTACAA
ATACTCATGGGTGGTATTCTTATTATTGGCTTCCATTTCTTCCACTAATTGTAATCTTATTAGTTGGTGCTAAGTTACAAATGATCA
TAACAAAAATGGGATTAAGGATTCAAGACAGAGGAGAAGTAATCAAGGGTGCACCTGTGGTTGAGCCTGGAGATCACCTTTTCT
GGTTCAATCGTCCTCACCTTCTTCTCTTCACGATTCATCTTGTTCTCTTTCAGAATGCCTTTCAACTTGCATTTTTTGCTTGGAGTAC
ATATGAGTTTTCCATAACCTCTTGCTTCCACAAAACAACTGCAGATAGTGTCATTAGAATCACTGTAGGGGTTGTAATACAAACTC
TATGTAGCTATGTGACTTTGCCTCTTTATGCTCTAGTCACACAGATGGGATCAACCATGAAACCAACCATTTTCAACGAAAGAGTG
GCAACAGCGCTTAAGAACTGGCACCACACAGCCAAAAAGCAGGTAAAACAGAGCAACCACTCAAACAACACGACACCGTATTCA
AGCAGGCCATCAACCCCAACACATGCCATGTCTCCTGTTCACCTGCTCCATAGACACACTGCTGGAAACAGCGACAGTCTACAAA
CTTCTCCGGAAAAGTCTGATTATAAAAATGAACAGTGGGATATTGAAGGAGAAGGACCAACTTCCCTAAGAAACGATCAAACAG
GGCAACATGAGATTCAAATAGCGGGTGTCGAGTCATTTTCGTCAACCGAATTGCCGGTTAGAATTAGACATGAAAGCACCTCTG
GTTCAAAAGATTTTTCTTTCGAGAAGCGCCACTTAGGGAGCAATTAGAATTGTAGGTATTGATAACCAGTTCAATGTATACCAATT
AGGTACATTCTTGCAGATAAAGATAGAGGAACTCCTTCTAAGAATGGAGTGTAAATTTGTTGAGGTAGCAGCTTGATTTGTGGA
TATAATCATAGGGTATGAAAATGCAAGACTATATTTTGTAAAAAAAAAAAA
>P-1287
TCTGGCTCTTCACAGTGCTTTTCCTTCTTACAAATACTCATGGGTGGTATTCTTATTATTGGCTTCCATTTCTTCCACTAATTGTAAT
CTTATTAGTTGGTGCTAAGTTACAAATGATCATAACAAAAATGGGATTAAGGATTCAAGACAGAGGAGAAGTAATCAAGGGTGC
ACCTGTGGTTGAGCCTGGAGATCACCTTTTCTGGTTCAATCGTCCTCACCTTCTTCTCTTCACGATTCATCTTGTTCTCTTTCAGAAT
GCCTTTCAACTTGCATTTTTTGCTTGGAGTACATATGAGTTTTCCATAACCTCTTGCTTCCACA
>P-1820
TCTGGCTCTTCACAGTGCTTTTCCTTCTTACAAATACTCATGGGTGGTATTCTTATTATTGGCTTCCATTTCTTCCACTAATTGTAAT
CTTATTAGTTGGTGCTAAGTTACAAATGATCATAACAAAAATGGGATTAAGGATTCAAGACAGAGGAGAAGTAATCAAGGGTGC
ACCTGTGGTTGAGCCTGGAGATCACCTTTTCTGGTTCAATCGTCCTCACCTTCTTCTCTTCACGATTCATCTTGTTCTCTTTCAGAAT
GCCTTTCAACTTGCATTTTTTGCTTGGAGTACATATGAGTTTTCCATAACCTCTTGCTTCCACA
>P-1516

TCTGGCTCTTCACAGTGCTTTTCCTTCTTACAAATACTCATGGGTGGTATTCTTATTATTGGCTTCCATTTCTTCCACTAATTGTAAT
CTTATTAGTTGGTGCTAAGTTACAAATGATCATAACAAAAATGGGATTAAGGATTCAAGACAGAGGAGAAGTAATCAAGGGTGC
ACCTGTGGTTGAGCCTGGAGATCACCTTTTCTGGTTCAATCGTCCTCACCTTCTTCTCTTCACGATTCATCTTGTTCTCTTTCAGAAT
GCCTTTCAACTTGCATTTTTTGCTTGGAGTACATATGAGTTTTCCATAACCTCTTGCTTCCACA
>P-144-10
TCTGGCTCTTCACAGTGCTTTTCCTTCTTACAAATACTCATGGGTGGTATTCTTATTATTGGCTTCCATTTCTTCCACTAATTGTAAT
CTTATTAGTTGGTGCTAAGTTACAAATGATCATAACAAAAATGGGATTAAGGATTCAAGACAGAGGAGAAGTAATCAAGGGTGC
ACCTGTGGTTGAGCCTGGAGATCACCTTTTCTGGTTCAATCGTCCTCACCTTCTTCTCTTCACGATTCATCTTGTTCTCTTTCAGAAT
GCCTTTCAACTTGCATTTTTTGCTTGGAGTACATATGAGTTTTCCATAACCTCTTGCTTCCACA
>P-1707
TCTGGCTCTTCACAGTGCTTTTCCTTCTTACAAATACTCATGGGTGGTATTCTTATTATTGGCTTCCATTTCTTCCACTAATTGTAAT
CTTATTAGTTGGTGCTAAGTTACAAATGATCATAACAAAAATGGGATTAAGGATTCAAGACAGAGGAGAAGTAATCAAGGGTGC
ACCTGTGGTTGAGCCTGGAGATCACCTTTTCTGGTTCAATCGTCCTCACCTTCTTCTCTTCACGATTCATCTTGTTCTCTTTCAGAAT
GCCTTTCAACTTGCATTTTTTGCTTGGAGTACATATGAGTTTTCCATAACCTCTTGCTTCCACA
>P-1610-9
TCTGGCTCTTCACAGTGCTTTTCCTTCTTACAAATACTCATGGGTGGTATTCTTATTATTGGCTTCCATTTCTTCCACTAATTGTAAT
CTTATTAGTTGGTGCTAAGTTACAAATGATCATAACAAAAATGGGATTAAGGATTCAAGACAGAGGAGAAGTAATCAAGGGTGC
ACCTGTGGTTGAGCCTGGAGATCACCTTTTCTGGTTCAATCGTCCTCACCTTCTTCTCTTCACGATTCATCTTGTTCTCTTTCAGAAT
GCCTTTCAACTTGCATTTTTTGCTTGGAGTACATATGAGTTTTCCATAACCTCTTGCTTCCACA
>P-1377
TCTGGCTCTTCACAGTGCTTTTCCTTCTTACAAATACTCATGGGTGGTATTCTTATTATTGGCTTCCATTTCTTCCACTAATTGTAAT
CTTATTAGTTGGTGCTAAGTTACAAATGATCATAACAAAAATGGGATTAAGGATTCAAGACAGAGGAGAAGTAATCAAGGGTGC
ACCTGTGGTTGAGCCTGGAGATCACCTTTTCTGGTTCAATCGTCCTCACCTTCTTCTCTTCACGATTCATCTTGTTCTCTTTCAGAAT
GCCTTTCAACTTGCATTTTTTGCTTGGAGTACATATGAGTTTTCCATAACCTCTTGCTTCCACA
>P-1506
TCTGGCTCTTCACAGTGCTTTTCCTTCTTACAAATACTCATGGGTGGTATTCTTATTATTGGCTTCCATTTCTTCCACTAATTGTAAT
CTTATTAGTTGGTGCTAAGTTACAAATGATCATAACAAAAATGGGATTAAGGATTCAAGACAGAGGAGAAGTAATCAAGGGTGC
ACCTGTGGTTGAGCCTGGAGATCACCTTTTCTGGTTCAATCGTCCTCACCTTCTTCTCTTCACGATTCATCTTGTTCTCTTTCAGAAT
GCCTTTCAACTTGCATTTTTTGCTTGGAGTACATATGAGTTTTCCATAACCTCTTGCTTCCACA
>DPMR-09-01
TCTGGCTCTTCACAGTGCTTTTCCTTCTTACAAATACTCATGGGTGGTATTCTTATTATTGGCTTCCATTTCTTCCACTAATTGTAAT
CTTATTAGTTGGTGCTAAGTTACAAATGATCATAACAAAAATGGGATTAAGGATTCAAGACAGAGGAGAAGTAATCAAGGGTGC
ACCTGTGGTTGAGCCTGGAGATCACCTTTTCTGGTTCAATCGTCCTCACCTTCTTCTCTTCACGATTCATCTTGTTCTCTTTCAGAAT
GCCTTTCAACTTGCATTTTTTGCTTGGAGTACATATGAGTTTTCCATAACCTCTTGCTTCCACA
>P-179
TCTGGCTCTTCACAGTGCTTTTCCTTCTTACAAATACTCATGGGTGGTATTCTTATTATTGGCTTCCATTTCTTCCACTAATTGTAAT
CTTATTAGTTGGTGCTAAGTTACAAATGATCATAACAAAAATGGGATTAAGGATTCAAGACAGAGGAGAAGTAATCAAGGGTGC
ACCTGTGGTTGAGCCTGGAGATCACCTTTTCTGGTTCAATCGTCCTCACCTTCTTCTCTTCACGATTCATCTTGTTCTCTTTCAGAAT
GCCTTTCAACTTGCATTTTTTGCTTGGAGTACATATGAGTTTTCCATAACCTCTTGCTTCCACA
>P-KPMR-400
TCTGGCTCTTCACAGTGCTTTTCCTTCTTACAAATACTCATGGGTGGTATTCTTATTATTGGCTTCCATTTCTTCCACTAATTGTAAT
CTTATTAGTTGGTGCTAAGTTACAAATGATCATAACAAAAATGGGATTAAGGATTCAAGACAGAGGAGAAGTAATCAAGGGTGC
ACCTGTGGTTGAGCCTGGAGATCACCTTTTCTGGTTCAATCGTCCTCACCTTCTTCTCTTCACGATTCATCTTGTTCTCTTTCAGAAT
GCCTTTCAACTTGCATTTTTTGCTTGGAGTACATATGAGTTTTCCATAACCTCTTGCTTCCACA
>P-1440-20
TCTGGCTCTTCACAGTGCTTTTCCTTCTTACAAATACTCATGGGTGGTATTCTTATTATTGGCTTCCATTTCTTCCACTAATTGTAAT
CTTATTAGTTGGTGCTAAGTTACAAATGATCATAACAAAAATGGGATTAAGGATTCAAGACAGAGGAGAAGTAATCAAGGGTGC
ACCTGTGGTTGAGCCTGGAGATCACCTTTTCTGGTTCAATCGTCCTCACCTTCTTCTCTTCACGATTCATCTTGTTCTCTTTCAGAAT
GCCTTTCAACTTGCATTTTTTGCTTGGAGTACATATGAGTTTTCCATAACCTCTTGCTTCCACA
>LFP-575
TCTGGCTCTTCACAGTGCTTTTCCTTCTTACAAATACTCATGGGTGGTATTCTTATTATTGGCTTCCATTTCTTCCACTAATTGTAAT
CTTATTAGTTGGTGCTAAGTTACAAATGATCATAACAAAAATGGGATTAAGGATTCAAGACAGAGGAGAAGTAATCAAGGGTGC
ACCTGTGGTTGAGCCTGGAGATCACCTTTTCTGGTTCAATCGTCCTCACCTTCTTCTCTTCACGATTCATCTTGTTCTCTTTCAGAAT
GCCTTTCAACTTGCATTTTTTGCTTGGAGTACATATGAGTTTTCCATAACCTCTTGCTTCCACA
>LFP-571

TCTGGCTCTTCACAGTGCTTTTCCTTCTTACAAATACTCATGGGTGGTATTCTTATTATTGGCTTCCATTTCTTCCACTAATTGTAAT
CTTATTAGTTGGTGCTAAGTTACAAATGATCATAACAAAAATGGGATTAAGGATTCAAGACAGAGGAGAAGTAATCAAGGGTGC
ACCTGTGGTTGAGCCTGGAGATCACCTTTTCTGGTTCAATCGTCCTCACCTTCTTCTCTTCACGATTCATCTTGTTCTCTTTCAGAAT
GCCTTTCAACTTGCATTTTTTGCTTGGAGTACATATGAGTTTTCCATAACCTCTTGCTTCCACA
>ACACIA
TCTGGCTCTTCACAGTGCTTTTCCTTCTTACAAATACTCATGGGTGGTATTCTTATTATTGGCTTCCATTTCTTCCACTAATTGTAAT
CTTATTAGTTGGTGCTAAGTTACAAATGATCATAACAAAAATGGGATTAAGGATTCAAGACAGAGGAGAAGTAATCAAGGGTGC
ACCTGTGGTTGAGCCTGGAGATCACCTTTTCTGGTTCAATCGTCCTCACCTTCTTCTCTTCACGATTCATCTTGTTCTCTTTCAGAAT
GCCTTTCAACTTGCATTTTTTGCTTGGAGTACATATGAGTTTTCCATAACCTCTTGCTTCCACA
>MR BIG
TCTGGCTCTTCACAGTGCTTTTCCTTCTTACAAATACTCATGGGTGGTATTCTTATTATTGGCTTCCATTTCTTCCACTAATTGTAAT
CTTATTAGTTGGTGCTAAGTTACAAATGATCATAACAAAAATGGGATTAAGGATTCAAGACAGAGGAGAAGTAATCAAGGGTGC
ACCTGTGGTTGAGCCTGGAGATCACCTTTTCTGGTTCAATCGTCCTCACCTTCTTCTCTTCACGATTCATCTTGTTCTCTTTCAGAAT
GCCTTTCAACTTGCATTTTTTGCTTGGAGTACATATGAGTTTTCCATAACCTCTTGCTTCCACA
>P-1280-4
TCTGGCTCTTCACAGTGCTTTTCCTTCTTACAAATACTCATGGGTGGTATTCTTATTATTGGCTTCCATTTCTTCCACTAATTGTAAT
CTTATTAGTTGGTGCTAAGTTACAAATGATCATAACAAAAATGGGATTAAGGATTCAAGACAGAGGAGAAGTAATCAAGGGTGC
ACCTGTGGTTGAGCCTGGAGATCACCTTTTCTGGTTCAATCGTCCTCACCTTCTTCTCTTCACGATTCATCTTGTTCTCTTTCAGAAT
GCCTTTCAACTTGCATTTTTTGCTTGGAGTACATATGAGTTTTCCATAACCTCTTGCTTCCACA
>P-1422-1
TCTGGCTCTTCACAGTGCTTTTCCTTCTTACAAATACTCATGGGTGGTATTCTTATTATTGGCTTCCATTTCTTCCACTAATTGTAAT
CTTATTAGTTGGTGCTAAGTTACAAATGATCATAACAAAAATGGGATTAAGGATTCAAGACAGAGGAGAAGTAATCAAGGGTGC
ACCTGTGGTTGAGCCTGGAGATCACCTTTTCTGGTTCAATCGTCCTCACCTTCTTCTCTTCACGATTCATCTTGTTCTCTTTCAGAAT
GCCTTTCAACTTGCATTTTTTGCTTGGAGTACATATGAGTTTTCCATAACCTCTTGCTTCCACA
>P-1660-2
TCTGGCTCTTCACAGTGCTTTTCCTTCTTACAAATACTCATGGGTGGTATTCTTATTATTGGCTTCCATTTCTTCCACTAATTGTAAT
CTTATTAGTTGGTGCTAAGTTACAAATGATCATAACAAAAATGGGATTAAGGATTCAAGACAGAGGAGAAGTAATCAAGGGTGC
ACCTGTGGTTGAGCCTGGAGATCACCTTTTCTGGTTCAATCGTCCTCACCTTCTTCTCTTCACGATTCATCTTGTTCTCTTTCAGAAT
GCCTTTCAACTTGCATTTTTTGCTTGGAGTACATATGAGTTTTCCATAACCTCTTGCTTCCACA
>P-1436-8
TCTGGCTCTTCACAGTGCTTTTCCTTCTTACAAATACTCATGGGTGGTATTCTTATTATTGGCTTCCATTTCTTCCACTAATTGTAAT
CTTATTAGTTGGTGCTAAGTTACAAATGATCATAACAAAAATGGGATTAAGGATTCAAGACAGAGGAGAAGTAATCAAGGGTGC
ACCTGTGGTTGAGCCTGGAGATCACCTTTTCTGGTTCAATCGTCCTCACCTTCTTCTCTTCACGATTCATCTTGTTCTCTTTCAGAAT
GCCTTTCAACTTGCATTTTTTGCTTGGAGTACATATGAGTTTTCCATAACCTCTTGCTTCCACA
>P-1806
TCTGGCTCTTCACAGTGCTTTTCCTTCTTACAAATACTCATGGGTGGTATTCTTATTATTGGCTTCCATTTCTTCCACTAATTGTAAT
CTTATTAGTTGGTGCTAAGTTACAAATGATCATAACAAAAATGGGATTAAGGATTCAAGACAGAGGAGAAGTAATCAAGGGTGC
ACCTGTGGTTGAGCCTGGAGATCACCTTTTCTGGTTCAATCGTCCTCACCTTCTTCTCTTCACGATTCATCTTGTTCTCTTTCAGAAT
GCCTTTCAACTTGCATTTTTTGCTTGGAGTACATATGAGTTTTCCATAACCTCTTGCTTCCACA
>P-1813
TCTGGCTCTTCACAGTGCTTTTCCTTCTTACAAATACTCATGGGTGGTATTCTTATTATTGGCTTCCATTTCTTCCACTAATTGTAAT
CTTATTAGTTGGTGCTAAGTTACAAATGATCATAACAAAAATGGGATTAAGGATTCAAGACAGAGGAGAAGTAATCAAGGGTGC
ACCTGTGGTTGAGCCTGGAGATCACCTTTTCTGGTTCAATCGTCCTCACCTTCTTCTCTTCACGATTCATCTTGTTCTCTTTCAGAAT
GCCTTTCAACTTGCATTTTTTGCTTGGAGTACATATGAGTTTTCCATAACCTCTTGCTTCCACA
>DMR-11
TCTGGCTCTTCACAGTGCTTTTCCTTCTTACAAATACTCATGGGTGGTATTCTTATTATTGGCTTCCATTTCTTCCACTAATTGTAAT
CTTATTAGTTGGTGCTAAGTTACAAATGATCATAACAAAAATGGGATTAAGGATTCAAGACAGAGGAGAAGTAATCAAGGGTGC
ACCTGTGGTTGAGCCTGGAGATCACCTTTTCTGGTTCAATCGTCCTCACCTTCTTCTCTTCACGATTCATCTTGTTCTCTTTCAGAAT
GCCTTTCAACTTGCATTTTTTGCTTGGAGTACATATGAGTTTTCCATAACCTCTTGCTTCCACA
>P-1804
TCTGGCTCTTCACAGTGCTTTTCCTTCTTACAAATACTCATGGGTGGTATTCTTATTATTGGCTTCCATTTCTTCCACTAATTGTAAT
CTTATTAGTTGGTGCTAAGTTACAAATGATCATAACAAAAATGGGATTAAGGATTCAAGACAGAGAAGAAGTAATCAAGGGAGC
ACAAATGGTTGATCCTGGCGATCACCTTTTCTGGATCCATCGTCCTCACCTTCTTCTCTTCACGATTCATCTTGTTCTCTTTCAGAAT
GCCTTTCAACTTGCATTTTTTGCTTGGAGAACATATGAGTTTTCCATAACCTCTTGCTTCCACA
>P-995

TCTGGCTCTTCACAGTGCTTTTCCTTCTTACAAATACTCATGGGTGGTATTCTTATTATTGGCTTCCATTTCTTCCACTAATTGTAAT
CTTATTAGTTGGTGCTAAGTTACAAATGATCATAACAAAAATGGGATTAAGGATTCAAGACAGAGGAGAAGTAATCAAGGGTGC
ACCTGTGGTTGAGCCTGGAGATCACCTTTTCTGGTTCAATCGTCCTCACCTTCTTCTCTTCACGAATCACCCTGTTCTCTTTCAGCAT
GCCTTTCAACTTGCATTTTTTGCTTGCAGTACATATGAgTTTTCCATAACCTCTTGCTTCCACA
>HFP-4
TCTGGCTCTTCACAGTGCTTTTCCTTCTTACAAATACTCATGGGTGGTATTCTTATTATTGGCTTCCATTTCTTCCACTAATTGTAAT
CTTATTAGTTGGTGCTAAGTTACAAATGATCATAACAAAAATGGGATTAAGGATTCAAGACAGAGGAGAAGTAATCAAGGGAGC
ACCTGTGGTTGAGCCTGGAGATCACCTTTTCTGGTTCAATCGTCCTCACCTTCTTCTCTTCACGATTCATCTTGTTCTCTTTCAGAAT
GCCTTTCAACTTGCATTTTTTGCTTGGAGTACATATGAGTTTTCCATAACCTCTTGCTTCCACA
>P-48
TCTGGCTCTTCACAGTGCTTTTCCTTCTTACAAATACTCATGGGTGGTATTCTTATTATTGGCTTCCATTTCTTCCACTAATTGTAAT
CTTATTAGTTGGTGCTAAGTTACAAATGATCATAACAAAAATGGGATTAAGGATTCAAGACAGAGGAGAAGTAATCAAGGGAGC
ACCTGTGGTTGAGCCTGGAGATCACCTTTTCTGGTTCAATCGTCCTCACCTTCTTCTCTTCACGATTCATCTTGTTCTCTTTCAGAAT
GCCTTTCAACTTGCATTTTTTGCTTGGAGTACATATGAGTTTTCCATAACCTCTTGCTTCCACA
>KMNR-894
TCTGGCTCTTCACAGTGCTTTTCCTTCTTACAAATACTCATGGGTGGTATTCTTATTATTGGCTTCCATTTCTTCCACTAATTGTAAT
CTTATTAGTTGGTGCTAAGTTACAAATGATCATAACAAAAATGGGATTAAGGATTCAAGACAGAGGAGAAGTAATCAAGGGAGC
ACCTGTGGTTGAGCCTGGAGATCACCTTTTCTGGTTCAATCGTCCTCACCTTCTTCTCTTCACGATTCATCTTGTTCTCTTTCAGAAT
GCCTTTCAACTTGCATTTTTTGCTTGGAGTACATATGAGTTTTCCATAACCTCTTGCTTCCACA
>P-668-1
TCTGGCTCTTCACAGTGCTTTTCCTTCTTACAAATACTCATGGGTGGTATTCTTATTATTGGCTTCCATTTCTTCCACTAATTGTAAT
CTTATTAGTTGGTGCTAAGTTACAAATGATCATAACAAAAATGGGATTGAGGATTCAAGACAGAGGAGAAGTAATCGAGGGGG
CAGAAGTGGTTGAGCCTGGAGATCACCTTTTCTGGTTCAATCGTCCTCACCTTCTTCTCTTCACGATTCATCTTGTTCTCTTTCAGA
ATGCCTTTCAACTTGCATTTTTTGCTTGGAGTACATATGAGTTTTCCATAACCTCTTGCTTCCACA
>P-1811
TCTGGCTCTTCACAGTGCTTTTCCTTCTTACAAATACTCATGGGTGGTATTCTTATTATTGGCTTCCATTTCTTCCACTAATTGTAAT
CTTATTAGTTGGTGCTAAGTTACAAATGATCATAACAAAAATGGGATTGAGGATTCAAGACAGAGGAGAAGTAATCAAGGGAG
CAGAAGTGGTTGAGCCTGGAGATCTCCTTTTCTGGTTCAATCGTCCTCACCTTCTTCTCTTCACGATTCATCTTGTTCTCTTTCAGA
ATGCCTTTCAACTTGCATTTTTTGCTTGGAGTACATATGAGTTTTCCATAACCTCTTGCTTCCACA
>IPF-99-25
TCTGGCTCTTCACAGTGCTTTTCCTTCTTACAAATACTCATGGGTGGTATTCTTATTATTGGCTTCCATTTCTTCCACTAATTGTAAT
CTTATTAGTTGGTGCTAAGTTACAAATGATCATAACAAAAATGGGATTAAGGATTCGAGACAGAGAAGAAGTAATGAAGGGAG
CCCCAGTTGTTGATCCTCTCGATCTTGTTTTCTGGATCCATCGTCCTCACCTTCTTCTCTTCACGAATCATCTTGTTCTCTTTCAGAAT
GCCTTTCAACTTGCATTTTTTGCTTGGAGTACATATGAGTTTTCCATAACCTCTTGCTTCCACA
>DPP-139-3
TCTGGCTCTTCACAGTGCTTTTCCTTCTTACAAATACTCATGGGTGGTATTCTTATTATTGGCTTCCATTTCTTCCACTAATTGTAAT
CTTATTAGTTGGTGCTAAGTTACAAATGATCATAACAAAAATGGGATTAAGGATTCAAGACAGAGGAGAAGTAATCAAGGGTGC
ACCTGTGGTTGAGCCTGGAGATCACCTTTTCTGGTTCAATCGTCCTCACCTTCTTCTCTTAACGTTCTAACTTGTTCTCTCACAGAAT
GCTTATTAGTTGGTGTTTTTTGCTTGGAGAACATAAGAGTTTTCCATAACCTCTTGCTTCCACA
>LFP-517
TCTGGCTCTTCACAGTGCTTTTCCTTCTTACAAATACTCATGGGTGGTATTCTTATTATTGGCTTCCATTTCTTCCACTAATTGTAAT
CTTATTAGTTGGTGCTAAGTTACAAATGATCATAACAAAAATGGGATTAAGGATTCAAGACAGAGGAGAAGTAATCAAGGGTGC
ACCTGTGGTTGAGCCTGGAGATCACCTTTTCTGGTTCAATCGTCCTCACCTTCTTCTAATCACGATCTAACTTGTTCTCTTAGAGAA
TGCTTTTTAGTTGGTGTTTTTTGCTTGGAGAACATAAGAGTTTTCCATAACCTCTTGCTTCCACA
>LFP-577
TCTGGCTCTTCACAGTGCTTTTCCTTCTTACAAATACTCATGGGTGGTATTCTTATTATTGGCTTCCATTTCTTCCACTAATTGTAAT
CTTATTAGTTGGTGCTAAGTTACAAATGATCATAACAAAAATGGGATTAAGGATTCGAGACAGAGAAGAAGCATTCGAGGGGG
CAGAAGTGGTTGAGCCTGAAGATCTTCTTTTCTGGATCAATCGTCCTCACCTTCTTCTCTTCACGATTCATCTTGTTCTCTTTCAGAA
TGCCTTTCAACTTGCATTTTTTGCTTGGAGTACATATGAGTTTTCCATAACCTCTTGCTTCCACA
>PB-29-B
TCTGGCTCTTCACAGTGCTTTTCCTTCTTACAAATACTCATGGGTGGTATTCTTATTATTGGCTTCCATTTCTTCCACTAATTGTAAT
CTTATTAGTTGGTGCTAAGTTACAAATGATCATAACAAAAATGGGATTAAGGATTCGAGACAGAGAAGAAGTACTCAAGGGAGC
ACCAGTGGTTGAGCCTGAAGATCACCTTTTCTGGTTCAATCGTCCTCACCTTCTTCTCTTCACGATTCATCTTGTTCTCTTTCAGAAT
GCCTTTCAACTTGCATTTTTTGCTTGGAGTACATATGAGTTTTCCATAACCTCTTGCTTCCACA
